# IGFBP1 Sustains Cell Survival during Spatially‐Confined Migration and Promotes Tumor Metastasis

**DOI:** 10.1002/advs.202206540

**Published:** 2023-06-09

**Authors:** Guoqing Cai, Yijun Qi, Ping Wei, Hong Gao, Chenqi Xu, Yun Zhao, Xiujuan Qu, Feng Yao, Weiwei Yang

**Affiliations:** ^1^ State Key Laboratory of Cell Biology Shanghai Key Laboratory of Molecular Andrology Shanghai Institute of Biochemistry and Cell Biology Center for Excellence in Molecular Cell Science Chinese Academy of Sciences University of Chinese Academy of Sciences Shanghai 200031 China; ^2^ Key Laboratory of Systems Health Science of Zhejiang Province School of Life Science Hangzhou Institute for Advanced Study University of Chinese Academy of Sciences Hangzhou 310024 China; ^3^ Department of Pathology Fudan University Shanghai Cancer Center Shanghai 200032 China; ^4^ State Key Laboratory of Molecular Biology, Shanghai Science Research Center, CAS Center for Excellence in Molecular Cell Science, Shanghai Institute of Biochemistry and Cell Biology Chinese Academy of Sciences shanghai 200031 China; ^5^ Department of Medical Oncology The First Hospital of China Medical University Shenyang 110001 China; ^6^ Department of Thoracic Surgery Shanghai Chest Hospital, Shanghai Jiao Tong University School of Medicine Shanghai 200031 China

**Keywords:** confined migration, insulin‐like growth factor‐binding protein 1, mitochondrial reactive oxygen species, superoxide dismutase, tumor metastasis

## Abstract

Cell migration is a pivotal step in metastatic process, which requires cancer cells to navigate a complex spatially‐confined environment, including tracks within blood vessels and in the vasculature of target organs. Here it is shown that during spatially‐confined migration, the expression of insulin‐like growth factor‐binding protein 1 (IGFBP1) is upregulated in tumor cells. Secreted IGFBP1 inhibits AKT1‐mediated phosphorylation of mitochondrial superoxide dismutase (SOD2) serine (S) 27 and enhances SOD2 activity. Enhanced SOD2 attenuates mitochondrial reactive oxygen species (ROS) accumulation in confined cells, which supports tumor cell survival in blood vessels of lung tissues, thereby accelerating tumor metastasis in mice. The levels of blood IGFBP1 correlate with metastatic recurrence of lung cancer patients. This finding reveals a unique mechanism by which IGFBP1 sustains cell survival during confined migration by enhancing mitochondrial ROS detoxification, thereby promoting tumor metastasis.

## Graphical abstract

An important in vivo migration mode is locomotion through confining spaces. In this study, the authors report that IGFBP1, a secreted protein, is upregulated in tumor cells during confined migration to promote metastasis. Mechanistically, secreted IGFBP1 activates mitochondrial superoxide dismutase (SOD2) by preventing AKT1‐mediated SOD2 phosphorylation, thereby promoting metastasis by sustaining tumor cell survival during confined migration.

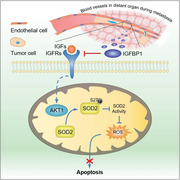

## 1 Introduction

Metastasis, the spread of cancerous cells from a primary tumor to distant sites, poses the biggest problem to cancer treatment and causes the majority of the death of cancer patients.^[^ ^1^ ^]^ The metastatic cascade is complex and encompasses the migration of cancer cells away from the primary tumor, the intravasation into the bloodstream or lymphatic system, the transport through the vessels, the extravasation to secondary tissues and the formation of distant metastatic tumor colonies.^[^ ^2^ ^]^ Cell migration is a pivotal step in such complex cascade and requires cancer cells to navigate a spatially complex microenvironment.^[^ ^3^ ^]^ An important in vivo migration mode is locomotion through confining spaces, such as the pores in extracellular matrix (ECM) of the tumor stroma^[^ ^4^ ^]^ or as tunnel‐like tracks, including tracks along ECM fibers in the interstitial space,^[^ ^5^ ^]^ between muscle and nerve fibers,^[^ ^6^ ^]^ along or within blood vessels^[^ ^7^ ^]^ and in the vasculature of target organs.^[^ ^8^ ^]^ Understanding the molecular mechanisms underlying the migration of cancer cells in confined spaces will accelerate the development of therapeutic interventions that halt the metastatic spread.

Cancer cells often rewire metabolic pathways to meet their increased bioenergetic and biosynthetic demands and mitigate oxidative stress arising from the increased metabolic activity associated with anabolic processes, which are required for cancer cell proliferation and survival.^[^ ^9^ ^]^ In contrast, less is known about the metabolic vulnerabilities of the cells that metastasize and colonize distal organs. Recently, emerging evidence indicate that metastatic potential is linked to the altered metabolism in cancer cells. For instance, knockdown of hexokinase 2 or pyruvate kinase M2, the two rate‐limiting glycolytic enzymes, results in reduced glycolysis and impairs the migration of breast cancer cells by regulating the by‐product methylglyoxal‐activated YAP signaling.^[^ ^10^ ^]^ In breast cancer, however, the expression of peroxisome proliferator‐activated receptor gamma coactivator 1‐alpha (PGC‐1*α*) drives oxidative metabolism as well as mitochondrial biogenesis, and in the final consequence, invasion leading to metastasis formation.^[^ ^11^ ^]^ Lipid accumulation and free fatty acid uptake increase the invasion, migration, and progression of breast and liver cancers, likely through the induction of epithelial–mesenchymal transition by transforming growth factor *β* and Wnt family member signaling.^[^ ^12^ ^]^ Additionally, metastatic melanoma cells upregulate the enzymes in oxidative phosphate pentose pathway to increase NADPH production, thereby elevating their antioxidant capacity, which allows circulating cancer cells to survive matrix detachment‐induced anoikis.^[^ ^13^ ^]^ However, which metabolic pathways are rewired in tumor cells during confined migration and how these rewired pathways promote cell migration in confining spaces and subsequent tumor metastasis remain unknown.

Insulin‐like growth factor‐binding protein 1 (IGFBP1) is a member of the family of secreted proteins that specifically binds and modulates the mitogenic and metabolic functions of insulin‐like growth factor (IGF)‐1 and IGF‐2.^[^ ^14^ ^]^ In this study, we carried out RNA‐sequencing analysis in tumor cells during confined migration and found that IGFBP1 is upregulated in confined cells and secreted IGFBP1 sustains cell survival during confined migration by regulating SOD2 phosphorylation and activity, thereby promoting tumor metastasis.

## 2 Results

### 2.1 IGFBP1 is Upregulated in Tumor Cells during Confined Migration

**Figure 1:**
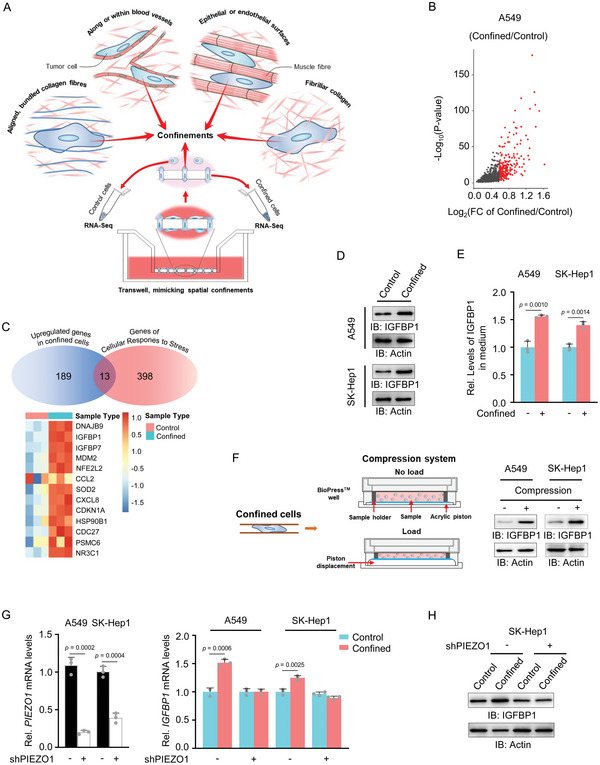
IGFBP1 is upregulated in tumor cells during confined migration. A) Schematic model for sample preparation of confined cells for RNA sequencing analysis. Transwell migration assay was performed in A549 lung cancer cells with a chamber of 8 µm pore size. The migrating cells were split into two groups. In control group, the cells did not through the pores of transwell inserts. In confined group, the cells were constrained in the pores. RNA‐sequencing (RNA‐Seq) analysis was performed in these two groups of the cells. B) Volcano plot shows upregulated genes (Fold change > 1, *p* < 0.05, FPKM > 10) in the confined cells. Highly upregulated genes (Fold change > 1.5, *p* < 0.05, FPKM > 10) were highlighted in red. C) A Venn diagram was used to visually represent overlapped 13 genes between upregulated genes in confined cells and genes of cellular response to stress pathway in Reactome database (top panel). Overlapped 13 genes were displayed in a heatmap. D) IGFBP1 expression was examined in A549 or SK‐Hep1 cells with or without confinement by using immunoblotting analyses. Brefeldin A (5 µm), inhibitor of protein secretion. E) A549 or SK‐Hep1 cells were cultured in Transwell chambers with 0.4 or 8 µm pore size. Culture media of the cells were collected for the examination of secreted IGFBP1 by ELISA assay. In the chambers of 0.4 µm pore size, the cells could not migrate through the pores and served as the control cells for confined cells. Data represent the mean ± S.D. of three independent experiments. F) Left is a model of compression system. Right is IGFBP1 protein levels in A549 or SK‐Hep1 cells with or without mechanical compression (5 kPa) were examined by immunoblotting analyses. G) The mRNA levels of PIEZO1 and IGFBP1 in A549 and SK‐Hep1 cells with or without PIEZO1 depletion in the absence or presence of confinement were detected by quantitative PCR analysis. H) The protein levels of IGFBP1 in SK‐Hep1 cells with or without PIEZO1 depletion in the absence or presence of confinement were detected by immunoblotting analyses. Immunoblots are representative of three independent experiments.

To identify the stress‐responsive regulator required for cell migration in spatial confinement, we adopted transwell migration system to simulate the confine migration of A549 lung cancer cells (**Figure** **1**A). The migrating cells were split into two groups, including control cells that have not passed through the pores of transwell chambers and confined cells that have been constrained in the pores. RNA‐sequencing (RNA‐Seq) analysis was performed in these two groups of the cells (Figure 1A). A volcano plot was generated to visualize the upregulated genes in confined cells (Figure 1B). We next intersected these upregulated genes with the genes in the category of genes of cellular response to stress pathway from the Reactome database and identified 13 overlapped genes, which were displayed in a heatmap (Figure 1C). The mRNA levels of top five changed genes were validated by quantitative PCR analysis (Figure S1A, Supporting Information). Importantly, among the five genes, only the expression of IGFBP1 correlated with the metastasis of lung cancer, as indicated in the analysis of RNA‐Seq data of lung adenocarcinoma from the cancer genome atlas (TCGA) (Figure S1B, Supporting Information). Similarly, the levels of IGFBP1 protein were also markedly increased in tumor cells during confined migration (Figure 1D). As IGFBP1 often functions as a secreted protein, we examined the secretion of IGFBP1 in confined cells by performing enzyme‐linked immunosorbent assay (ELISA), showing that IGFBP1 secretion was also markedly induced by the confinement (Figure 1E).

Mechanical forces in tumor microenvironment may arise due to excessive growth of cells in a confinement as in case of solid tumors, interstitial flows within tumors and due to blood flow in the vasculature as in case of circulating tumor cells.^[^ ^15^ ^]^ Thus, we tested whether mechanical force is responsible for the upregulation of IGFBP1 in confined cells. We adopted a compression device to apply mechanical force on cells and examined IGFBP1 expression in the cells subjected to mechanical compression, which showed that IGFBP1 expression is dramatically increased in tumor cells after compression (Figure 1F). In addition, we depleted PIEZO1, a well‐established mechanosensitive channel, in tumor cells and observed that PIEZO1 depletion abrogated confinement‐induced upregulation of IGFBP1 mRNA and protein levels (Figure 1G,H). Conversely, PIEZO1 activation by Yoda1, a selective activator of PIEZO1, increased IGFBP1 protein levels (Figure S1C, Supporting Information). The activation of PIEZO1 normally activates the downstream signal transduction through Ca^2+^ signals.^[^ ^16^ ^]^ To further determine whether PIEZO1 regulates IGFBP1 expression through Ca^2+^ signaling. We treated IGFBP1‐depleted cells or control cells with or without compression in the absence or presence of Ca^2+^ chelator EGTA acetoxymethyl ester (EGTA‐AM). Immunoblotting assay showed that either PIEZO1 depletion or EGTA‐AM treatment abrogated compression‐induced upregulation of IGFBP1 in SK‐Hep1 cells, while PIEZO1 depletion combined with EGTA‐AM treatment did not further decrease IGFBP1 expression (Figure S1D, Supporting Information), suggesting that PIEZO1 increases IGFBP1 expression through Ca^2+^ signaling. Of note, IGFBP1 depletion did not influence PIEZO1 expression in confined tumor cells (Figure S1E, Supporting Information).

Collectively, these results demonstrate that the expression and secretion of IGFBP1 were increased in tumor cells during confined migration at least partly in response to mechanical compression.

### 2.2 IGFBP1 Expression Correlates with Metastatic Recurrence and Prognosis of Lung Cancer Patients

To define the relationship between the expression of IGFBP1 and the survival duration of lung cancer patients, we performed survival analysis of lung adenocarcinoma patients by using UALCAN database (http://ualcan.path.uab.edu), which showed that the patients whose tumors had high expression of IGFBP1 (124 cases) exhibited shorter survival time than those with lower expression of IGFBP1 (378 cases) in the tumor cells (Figure S1F, Supporting Information). Moreover, immunohistochemistry (IHC) analysis of IGFBP1 expression in primary tumors from lung adenocarcinoma patients with or without metastatic recurrence further confirmed that the tumors from the patients with recurrence had higher expression of IGFBP1 than those from the patients without (Figure S1G, Supporting Information). The specificity of anti‐IGFBP1 antibody for IHC analysis was validated by IGFBP1 recombinant protein blocking assay (Figure S1H, Supporting Information). In addition, we carried out ELISA assay to examine IGFBP1 protein levels in blood samples from lung adenocarcinoma patients and compared blood IGFBP1 levels between the patients with or without metastatic recurrence, which showed that the patients with recurrence had higher IGFBP1 levels in blood than those without (Figure S1I, Supporting Information). We then evaluated the ability of blood IGFBP1 levels to distinguish between the patients with metastatic and non‐metastatic tumors by performing receiver operator‐characteristic curve (ROC) analysis, implicating that blood IGFBP1 could potentially be used as a diagnostic biomarker for metastatic lung cancer (Figure S1J, Supporting Information).

### 2.3 IGFBP1 Is Required for Tumor Cell Migration and Metastasis In Vivo

**Figure 2:**
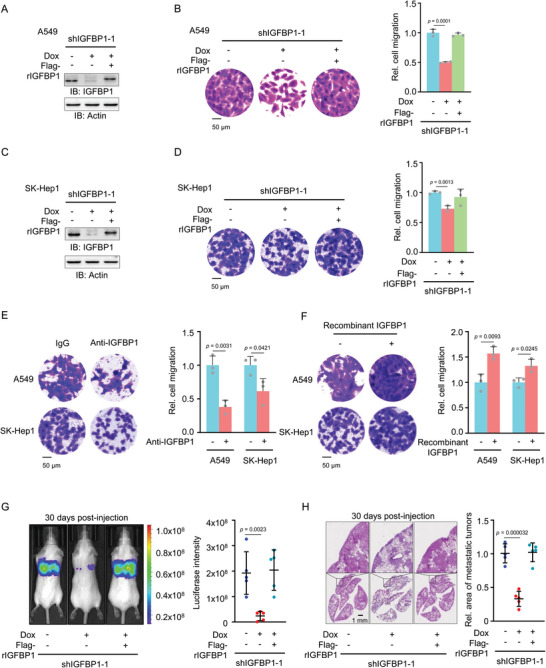
IGFBP1 is required for tumor cell migration and metastasis in vivo. A,B) IGFBP1‐depleted A549 cells were rescued with or without Flag‐rIGFBP1. IGFBP1 expression was examined by immunoblotting analyses (A). Transwell migration assays were performed. Representative images (left panel) and statistical analysis (right panel) of the migrated cells was shown (B). Relative cell migration was normalized to A549 cells without IGFBP1 depletion. C,D) IGFBP1‐depleted SK‐Hep1 cells was rescued with Flag‐rIGFBP1. IGFBP1 expression was examined by immunoblotting analyses (C). Transwell migration assays were performed (D). Relative cell migration was normalized to SK‐Hep1 cells without IGFBP1 depletion. E) A549 or SK‐Hep1 cells were treated with IgG or anti‐IGFBP1 antibody. Transwell migration assays were performed. F) A549 or SK‐Hep1 cells were treated with or without recombinant IGFBP1. Transwell migration assays were performed. G,H) Luciferase‐expressing IGFBP1‐depleted A549 cells rescued with Flag‐rIGFBP1 were injected into randomized NOD/SCID mice by tail vein injection (five mice per group). After 30 days of inoculation, bioluminescence imaging was performed and representative images of lung metastasis were presented (G, left panel). The statistical analysis of luciferase intensities was shown (G, right panel). After bioluminescence imaging, mice were euthanized. Representative images of H&E staining of lung sections from these mice were shown (H, left panel). Tumor areas in H&E‐stained sections were calculated and normalized to those of the mice injected with A549 cells without IGFBP1 depletion (H, right panel). Data represent the mean ± S.D. of five mice. (B, D–F), Data represent the mean ± S.D. of three independent experiments.

To determine the role of IGFBP1 in tumor cell migration, we depleted endogenous IGFBP1 with shRNA in A549 lung adenocarcinoma cells and rescued the cells with shRNA‐resistant (r) IGFBP1 (**Figure** **2**A). We performed transwell migration assay with these cells, which showed that IGFBP1 depletion dramatically inhibited the migration of these cells and rescued expression of Flag‐rIGFBP1 almost completely recovered the migratory capability of IGFBP1‐depleted cells (Figure 2B), excluding the off‐target possibility of IGFBP1 shRNA. Similar results were also obtained in IGFBP1‐depleted SK‐Hep1 liver cancer cells rescued with Flag‐rIGFBP1 (Figure 2C,D). The levels of secreted IGFBP1 were also examined, showing that IGFBP1 depletion decreased the levels of secreted IGFBP1, while the expression of rescued Flag‐rIGFBP1 recovered the levels of secreted IGFBP1 to control cells (Figure S2A, Supporting Information). To clarify whether secreted IGFBP1 is responsible for the migration of tumor cells, we neutralized IGFBP1 in culture medium with anti‐IGFBP1 antibody and examined the migration of A549 or SK‐Hep1 cells by using transwell migration assay, which showed that IGFBP1 neutralization dramatically attenuated the migration of these cells (Figure 2E).

In addition, we examined the level of IGFBP1 protein in A549 and SK‐Hep1 and observed that A549 cells expressed lower IGFBP1 protein than SK‐Hep1 cells (Figure S2B, Supporting Information). Thus, we overexpressed IGFBP1 in A549 cells (Figure S2C, Supporting Information) and found that IGFBP1 overexpression dramatically promoted the migration of these cells (Figure S2D, Supporting Information). Moreover, we expressed and purified recombinant IGFBP1 protein and incubated A549 and SK‐Hep1 cells with or without recombinant IGFBP1. The migratory capability of both cells was markedly enhanced by IGFBP1 incubation (Figure 2F). The levels of IGFBP1 in culture medium of A549 and SK‐Hep1 cells were also validated (Figure S2E, Supporting Information).

We next investigated the role of IGFBP1 in tumor metastasis in vivo by using lung metastasis mouse model via tail vein injection. In the model, luciferase‐expressing IGFBP1‐depleted A549 cells rescued with or without Flag‐rIGFBP1 were injected via tail vein into 6 weeks old randomized female NOD/SCID mice. Bioluminescence imaging of the mice showed that the depletion of IGFBP1 dramatically impaired lung metastasis of the cells, while restored expression of Flag‐rIGFBP1 almost completely rescued tumor metastasis of IGFBP1‐depleted cells (Figure 2G). This result was further evidenced by hematoxylin eosin (H&E) staining of dissected lung tissues (Figure 2H). The level of IGFBP1 was also validated in the metastatic tumors by IHC staining (Figure S2F, Supporting Information). Taken together, these results demonstrate that secreted IGFBP1 is required for tumor cell migration and subsequent tumor metastasis.

### 2.4 IGFBP1 Promotes Tumor Metastasis by Sustaining Tumor Cell Survival during Confined Migration

**Figure 3:**
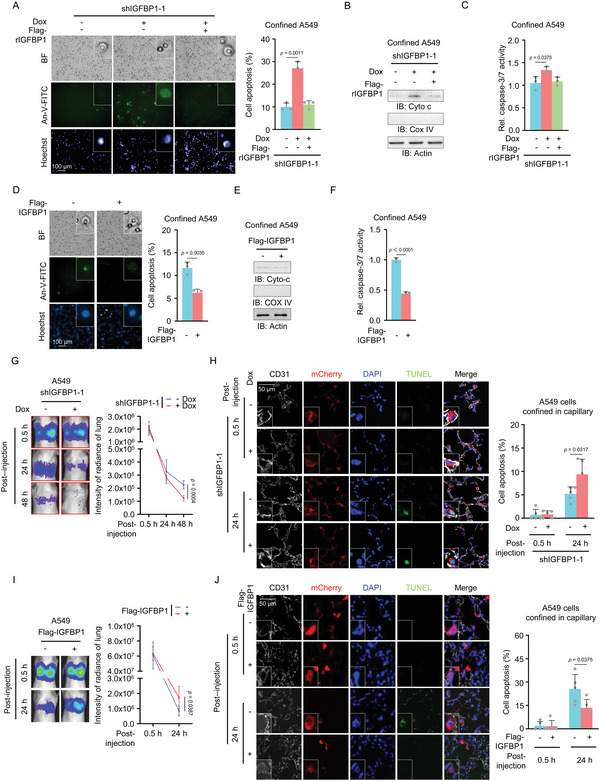
IGFBP1 promotes tumor metastasis by sustaining cell survival during confined migration. A–C) IGFBP1‐depleted A549 cells were rescued with Flag‐rIGFBP1. Transwell migration assays were performed. After 6 h of culture, the confined cells were stained with Annexin‐V‐FITC and photographed in situ. Representative images of the apoptotic confined cells were presented (A, left panel). The percentages of apoptosis of confined cells were shown (A, right panel). Data represent the mean ± S.D. of three independent experiments. (B) The confined cells were harvested and subjected to cell fractionation assay. The cytoplasmic proteins were used for immunoblotting analyses. (C) Caspase‐3/7 activities of confined cells were examined with the caspase‐3/7 activity detection kit. Relative caspase‐3/7 activities were normalized to those of A549 cells without IGFBP1 depletion. Data represent the mean ± S.D. of three independent experiments. D–F) Transwell migration assays were performed in A549 cells with or without IGFBP1 overexpression. After 6 h of culture, the confined cells were stained with Annexin‐V‐FITC and photographed in situ. Representative images of the apoptotic confined cells were presented (D, left panel). The percentages of apoptosis of confined cells were shown (D, right panel). Data represent the mean ± S.D. of three independent experiments. (E) The confined cells were harvested and subjected to cell fractionation assay. The cytoplasmic proteins were used for immunoblotting analyses. (F) Caspase‐3/7 activities of confined cells were examined with the caspase‐3/7 activity detection kit. Relative caspase‐3/7 activities were normalized to those of A549 cells without IGFBP1 overexpression. Data represent the mean ± S.D. of three independent experiments. G) Luciferase‐expressing A549 cells with or without IGFBP1 depletion were injected into the tail vein of NOD/SCID mice (five mice per group). Bioluminescence imaging of these mice were performed at indicated time point and representative images of tumor cell signals in lung were shown (left panel). Data represent the mean ± S.D. of luciferase intensities from five mice (right panel). H) mCherry‐expressing A549 cells with or without IGFBP1 depletion were injected into NOD/SCID mice (five mice per group) via tail vein, and lungs of the mice were dissected at 0.5 or 24 h after injection. TUNEL staining and IF staining with anti‐CD31 antibody of the lung sections were performed. Representative images of apoptotic cells in blood vessels were shown (left panel). Percentages of apoptotic cells were calculated and shown (right panel). Data represent the mean ± S.D. of five mice. I) Luciferase‐expressing A549 cells with or without IGFBP1 overexpression were injected into the tail vein of NOD/SCID mice (five mice per group). Bioluminescence imaging of these mice were performed at indicated time point and representative images of tumor cell signals in lung were shown (left panel). Data represent the mean ± S.D. of luciferase intensities from five mice (right panel). J) mCherry‐expressing A549 cells with or without IGFBP1 overexpression were injected into NOD/SCID mice (five mice per group) via tail vein, and lungs of the mice were dissected at 0.5 or 24 h after injection. TUNEL staining and IF staining with anti‐CD31 antibody of the lung sections were performed. Representative images of apoptotic cells in blood vessels were shown (left panel). Percentages of apoptotic cells were calculated and shown (right panel). Data represent the mean ± S.D. of five mice.

To explore the mechanism of IGFBP1‐promoted tumor cell migration, we performed wound healing assay with A549 cells with or without IGFBP1 depletion, which showed that IGFBP1 depletion did not influence the mobility of these cells (Figure S3A, Supporting Information). Similarly, the proliferation of these cells was not influenced by IGFBP1 depletion either (Figure S3B, Supporting Information). We then tested whether IGFBP1 influenced the viability of the cells confined in the pores of transwell chambers. To test the hypothesis, we performed transwell migration assay with A549 or SK‐Hep1 cells with or without IGFBP1 depletion, followed by the analysis of apoptosis in confined cells using Annexin‐V‐FITC staining. IGFBP1 depletion markedly increased the apoptosis of the cells in confining pores, while rescued expression of Flag‐rIGFBP1 recovered the survival of the cells (**Figure** **3**A and Figure S3C, Supporting Information). Moreover, the confined cells were harvested for the examination of cytochrome c in cytosol, which showed that IGFBP1 depletion markedly increased the levels of cytosolic cytochrome c in confined cells (Figure 3B and Figure S3D, Supporting Information). The assay of caspase 3/7 activity also confirmed that IGFBP1 depletion increased the apoptosis of confined cell (Figure 3C and Figure S3E, Supporting Information). In contrast, IGFBP1 depletion did not influence cell apoptosis, cytochrome c release, and caspase 3/7 activation in tumor cells without spatial constraints (Figure S3F–J, Supporting Information). In addition, IGFBP1 overexpression dramatically decreased the apoptosis of confined cells, as indicated by Annexin V staining, cytochrome c release, and caspase 3/7 activity assays (Figure 3D–F). These results indicate that IGFBP1 specifically sustains tumor cell survival during confined migration without influencing the mobility and proliferation of the cells.

Locomotion through confining spaces, including along or within blood vessels^[^ ^7^ ^]^ and in the vasculature of target organs^[^ ^8a^ ^]^ is the important in vivo migration mode. To determine whether IGFBP1 contributes to tumor cell survival in the vasculature of lung tissues during metastasis, we implanted luciferase‐expressing A549 cells with or without IGFBP1 into NOD/SCID mice via tail vein injection and monitored tumor cells in lung tissues in real time. Bioluminescence imaging of the mice showed that IGFBP1 depletion accelerated the diminishment of bioluminescence signals from tumor cells in lung tissues (Figure 3G). Moreover, we sacrificed these mice and dissected lung tissues at 0.5 or 24 h after injection. TUNEL assay was performed in these lung tissues, showing that IGFBP1 depletion dramatically increased the percentage of apoptotic cells in blood vessels of lung tissues (Figure 3H). IGFBP1 protein levels were validated in the cells confined in blood vessels by immunofluorescence staining (Figure S3K, Supporting Information). On the contrary, IGFBP1 overexpression slowed down the diminishment of tumor cell signals in lung and dramatically decreased the percentage of apoptotic cells in blood vessels of lung tissues (Figure 3I,J). Taken together, these results suggest that IGFBP1 promotes tumor metastasis by inhibiting cell apoptosis in blood vessels of lung tissues.

### 2.5 IGFBP1 Sustains the Survival of Confined Cells by Scavenging Mitochondrial ROS

**Figure 4:**
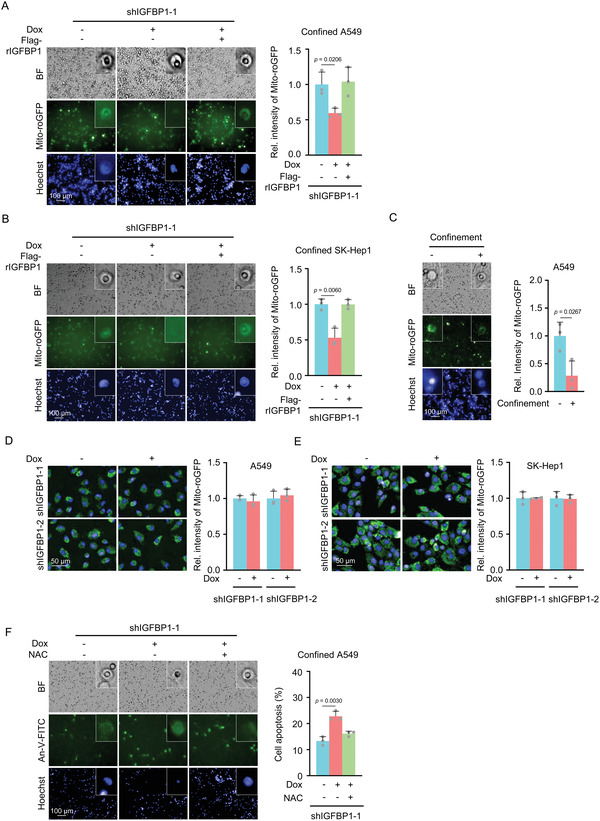
IGFBP1 sustains the survival of confined cells by scavenging mitochondrial ROS. A,B) Transwell migration assays were performed in Mito‐roGFP‐expressing IGFBP1‐depleted A549 (A) or SK‐Hep1 (B) cells rescued with Flag‐rIGFBP1. After 6 h of culture, cells were photographed. Representative images of Mito‐roGFP in confined cells were presented (left panel). Mito‐roGFP fluorescence intensities of confined cells were normalized to those of the Mito‐roGFP‐expressing cells without IGFBP1 depletion and were shown (right panel). C) Transwell migration assays were performed in Mito‐roGFP‐expressing A549 cells. After 6 h of culture, cells were photographed in situ. Representative images of Mito‐roGFP in cells with or without confinement were presented (left panel). Mito‐roGFP fluorescence intensities of cells with or without confinement were normalized to those of the Mito‐roGFP‐expressing cells without confinement and were shown (right panel). D,E) Mito‐roGFP‐expressing A549 (D) or SK‐Hep1 (E) cells with or without IGFBP1 depletion were seeded in the 96‐well plates. After 24 h of culture, the cells were photographed and representative images of Mito‐roGFP were presented (top panel). Relative Mito‐roGFP fluorescence intensities were normalized to those of Mito‐roGFP‐expressing cells without IGFBP1 depletion (bottom panel). F) IGFBP1‐depleted A549 cells were rescued with Flag‐rIGFBP1. The cells were treated with or without NAC (5 mm). Transwell migration assays were performed. After 6 h of culture, the cells were stained with Annexin‐V‐FITC and photographed in situ. Representative images of the apoptotic confined cells were presented (left panel). The percentages of apoptosis of confined cells were shown on the right panel. (A–F), Data represent the mean ± S.D. of three independent experiments.

Accumulating evidence show that a variety of physical force stimuli will give rise to reactive oxygen species (ROS),^[^ ^17^ ^]^ which is unevenly distributed in the cells.^[^ ^18^ ^]^ Excessive ROS causes cell apoptosis.^[^ ^19^ ^]^ To determine whether IGFBP1 promotes the survival of confined cells by scavenging excessive ROS, we measure the level of ROS in spatially‐confined tumor cells with or without IGFBP1 depletion by using the genetically‐encoded fluorescent biosensors, mitochondria‐targeted redox sensing GFP (mito‐roGFP) and cytosol‐targeted redox sensing GFP (cyto‐roGFP). The efficiency and accuracy of these two redox sensors were validated by the treatment of tert‐butyl hydroperoxide (tBH), a lipid‐soluble organic peroxide, and dithiothreitol (DTT), a reducing agent, showing that tBH treatment decreased the intensities of mito‐roGFP and cyto‐roGFP, while DTT treatment increased the intensities of both sensors (Figure S4A,B, Supporting Information). Of note, high levels of ROS resulted in low intensities of the sensors. Thus, as shown in **Figure** **4**A,B, IGFBP1 depletion dramatically increased mitochondrial ROS levels in confined cells, while rescued expression of Flag‐rIGFBP1 restored mitochondrial ROS levels in the cells. In contrast, the levels of ROS in the cytosol were not influenced by IGFBP1 depletion in confined cells (Figure S4C,D, Supporting Information). Of note, the confined A549 cells had much higher mitochondrial ROS levels than the cells under no confinement (Figure 4C). And in the cells under no confinement, the levels of mitochondrial ROS were not influenced by IGFBP1 depletion (Figure 4D,E). Then we treated the cells with antioxidant N‐acetyl‐L‐cysteine (NAC) and observed it greatly recovered the viability of IGFBP1‐depleted cells in confinement (Figure 4F). However, under no confinement, NAC treatment did not influence tumor cell apoptosis (Figure S4E, Supporting Information). These results demonstrate that scavenging mitochondrial ROS is required for IGFBP1‐promoted tumor cell survival in confinement.

### 2.6 IGFBP1 Scavenges Mitochondrial ROS by Activating SOD2

**Figure 5:**
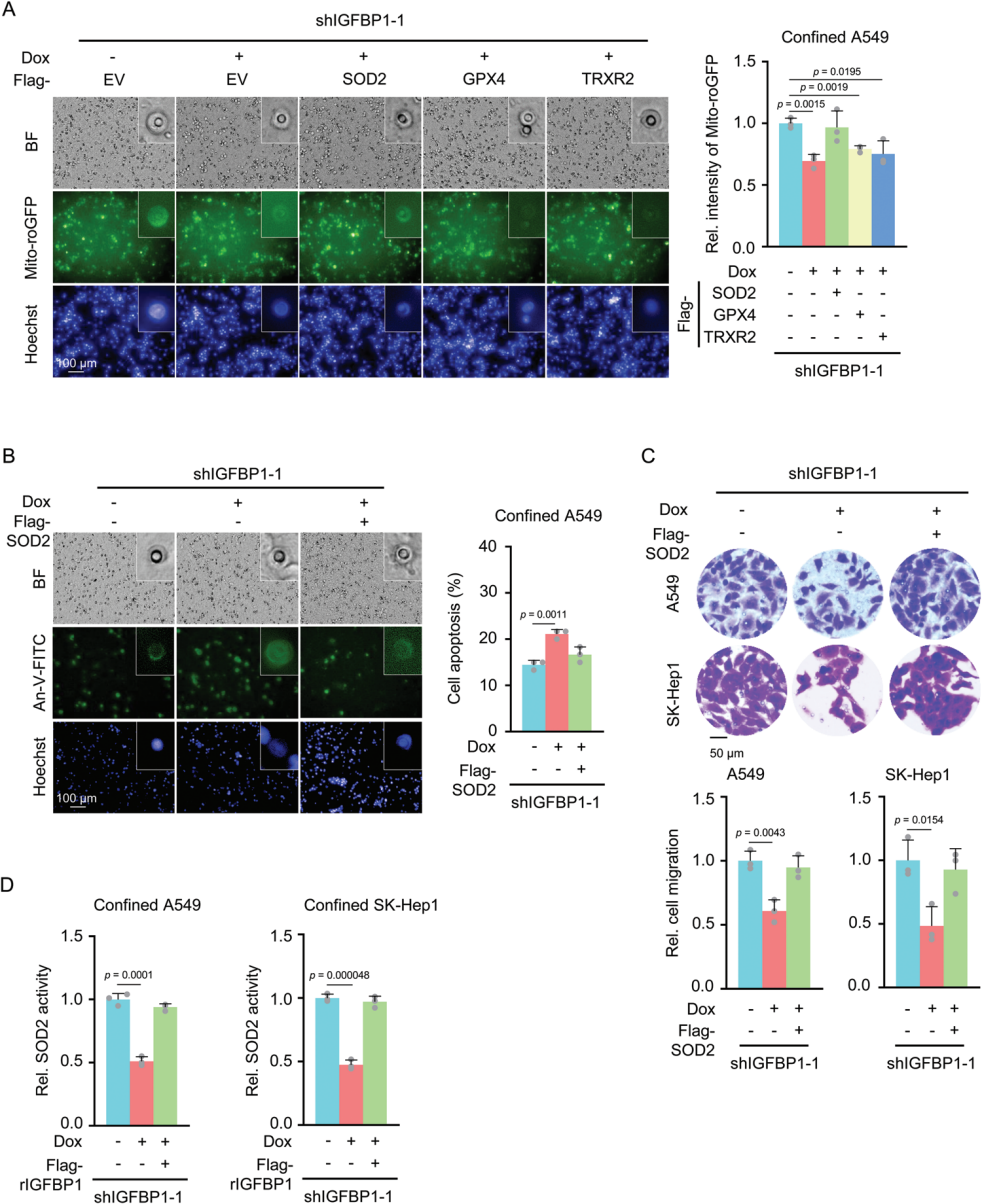
IGFBP1 scavenges mitochondrial ROS by activating SOD2. A) Mito‐roGFP‐expressing IGFBP‐depleted A549 cells were overexpressed with empty vector (EV), SOD2, GPX4, or TRXR2. Transwell migration assay was performed. After 6 h of culture, cells were photographed. Representative images of Mito‐roGFP in confined cells were presented (left panel). Mito‐roGFP fluorescence intensities of confined cells were normalized to those of the Mito‐roGFP‐expressing cells without IGFBP1 depletion and shown on the right panel. B) Mito‐roGFP‐expressing IGFBP‐depleted A549 cells were overexpressed with EV or SOD2. Transwell migration assays were performed. After 6 h of culture, cells were stained with Annexin‐V‐FITC and photographed in situ. Representative images of the apoptotic confined cells are presented (left panel). The percentages of apoptosis of confined cells were shown on the right panel. C) IGFBP1‐depleted A549 and SK‐Hep1 cells were overexpressed with EV or SOD2. Transwell migration assays were performed. D) IGFBP1‐depleted A549 and SK‐Hep1 cells were overexpressed with EV or SOD2. Transwell migration assays were performed. After 6 h of culture, SOD2 activities of confined cells were measured by WST‐8 kit (Beyotime Biotechnology). Relative SOD2 activities were normalized to the cells without IGFBP1 depletion. (A–D), Data represent the mean ± S.D. of three independent experiments.

Since mitochondria are the major sites of free radical generation, they are highly enriched with antioxidants including glutathione (GSH) and enzymes, such as superoxide dismutase 2 (SOD2), glutathione peroxidase (GPX4), and thioredoxin reductase 2 (TRXR2).^[^ ^20^ ^]^ To explore the underlying mechanism of IGFBP1‐sustained ROS homeostasis, we overexpressed SOD2, GPX4, or TRXR2 in IGFBP1‐depleted A549 cells (Figure S5A, Supporting Information) and observed that overexpression of SOD2, but not the others, reduced mitochondrial ROS levels and increased the viability in IGFBP1‐depleted cells in confined spaces (**Figure** **5**A,B). The levels of endogenous SOD2 were also examined in IGFBP1‐depleted A549 cells with or without SOD2 overexpression (Figure S5B, Supporting Information). Consequently, the migratory capability of IGFBP1‐depleted A549 or SK‐Hep1 cells was also recovered by SOD2 overexpression (Figure 5C). These results indicate that SOD2 is required for IGFBP1‐promoted mitochondrial ROS hemostasis and the survival and migration of confined cells.

We next explored how IGFBP1 regulated SOD2. Immunoblotting analysis showed that IGFBP1 depletion did not influence SOD2 protein levels in confined A549 cells (Figure S5C, Supporting Information). Instead, the enzymatic activity of SOD2 was markedly decreased in IGFBP1‐depleted A549 and SK‐Hep1 cells, which could be restored by rescued expression of Flag‐rIGFBP1 in the cells (Figure 5D and Figure S5D, Supporting Information). In contrast, SOD2 activities were not affected by IGFBP1 depletion in the cells under no confinement (Figure S5E, Supporting Information). These results indicate that IGFBP1 regulates SOD2 activity without influencing its protein levels.

### 2.7 IGFBP1 Enhances SOD2 Activity by Preventing AKT1‐Mediated SOD2 S27 Phosphorylation

**Figure 6:**
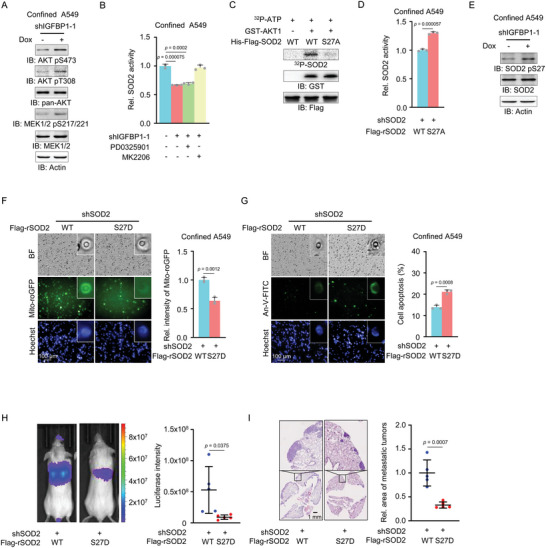
IGFBP1 enhances SOD2 activity by inhibiting AKT1‐dependent SOD2 pS27 and promotes tumor metastasis. A) Transwell migration assays of A549 cells with or without IGFBP1 depletion were performed. After 6 h of culture, the confined cells were collected and subjected to immunoblotting analyses with indicated antibodies. B) A549 cells with or without IGFBP1 depletion were treated with or without the inhibitor of MEK (PD0325901, 10 µm) or AKT1/2/3 (MK2206, 5 µm), followed by Transwell migration assays. After 6 h of culture, SOD2 activities in confined cells were measured by WST‐8 kit. Relative SOD2 activities were normalized to the cells without IGFBP1 depletion. C) In vitro kinase assay was performed by mixing bacterial‐ purified recombinant GST‐AKT1 and recombinant WT or S27A mutant SOD2. D) Transwell migration assays of SOD2‐depleted A549 cells rescued with Flag‐rSOD2 WT or S27A were performed. After 6 h of culture, SOD2 activities in confined cells were measured by WST‐8 kit. Relative SOD2 activities were normalized to rSOD2 WT. E) Transwell migration assays of A549 cells with or without IGFBP1 were performed. After 6 h of culture, SOD2 S27 phosphorylations of confined cells were detected. F) Mito‐roGFP‐expressing SOD2‐depleted A549 cells were rescued with WT or S27D mutant SOD2. Transwell migration assays were performed. Representative images of Mito‐roGFP in confined cells were presented (left panel). Mito‐roGFP fluorescence intensities of confined cells were normalized to those of the cells expressing rSOD2 WT (right panel). G) SOD2‐depleted A549 cells were rescued with WT or S27D mutant SOD2. Transwell migration assays were performed. After 6 h of culture, the cells were stained with Annexin‐V‐FITC and photographed in situ. Representative images of the apoptotic confined cells were presented on the left panel. The percentages of apoptosis of confined cells were shown on the right panel. H,I) Luciferase‐expressing SOD2‐depleted A549 cells rescued with WT or S27D mutant SOD2 were injected into randomized NOD/SCID mice by tail vein injection (five mice per group). After 30 days inoculation, bioluminescence imaging was performed and representative images of lung metastasis were presented (right panel). The statistical analysis of luciferase intensities was shown respectively on the left panel (H). Data represent the mean ± S.D. of five mice. After bioluminescence imaging, mice were euthanized. Representative images of H&E staining of lung sections from these mice were shown (I, right). Tumor areas in H&E‐stained sections were calculated and normalized to those of the mice injected with A549 cells expressing WT SOD2 (I, left). Data represent the mean ± S.D. of five mice. (B, D, F, G), Data represent the mean ± S.D. of three independent experiments.

As PI3K/AKT and MEK/ERK signaling pathways are the two major pathways downstream IGFs signaling,^[^ ^21^ ^]^ to gain insight into the mechanism underlying IGFBP1‐enhanced SOD2 activity, we examined the activation of AKT1, reported to translocate into mitochondria after the phosphorylation at serine (S) 473 and threonine (T) 308 stimulated by IGF‐1,^[^ ^22^ ^]^ and MEK1/2. Immunoblotting analysis indicated that the phosphorylation of AKT1 at S473 or T308 and the phosphorylation of MEK1/2 at S217/221 were increased after IGFBP1 depletion in confined cells (**Figure** **6**A). To identify which pathway is responsible for the regulation of SOD2 activity, we treated IGFBP1‐depleted cells with MEK inhibitor (PD0325901) and AKT inhibitor (MK2206) respectively. The efficacies of these inhibitors were validated by the phosphorylation of ERK1/2 tyrosine (Y) 204 and AKT S473 (Figure S6A, Supporting Information). As shown in Figure 6B, the treatment of MK2206, but not PD0325901, restored the activity of SOD2 in IGFBP1‐depleted cells in confined space, suggesting that IGFBP1 enhances SOD2 activity by inhibiting AKT1 in confined cells. Of note, IGFBP1 depletion did not influence AKT1 activation, indicated by AKT1 S473 and T308 phosphorylation without spatial confinement (Figure S6B, Supporting Information).

To uncover the mechanism of AKT1‐suppressed SOD2 activity, we first tested whether AKT1 phosphorylates SOD2. In vitro kinase assay was performed by mixing commercial recombinant GST‐AKT1 and bacterial‐purified His‐Flag‐SOD2, followed by mass spectrometry analysis of SOD2 phosphorylation, which showed that AKT1 phosphorylated SOD2 at S27 (Figure S6C,D, Supporting Information). The activity of GST‐AKT1 was validated by in vitro kinase assay by mixing GST‐AKT1 and HA‐S6K immunoprecipitated from HEK293T cells, followed by immunoblotting analysis of S6K T389 phosphorylation (Figure S6E, Supporting Information). AKT1‐dependent SOD2 S27 phosphorylation (SOD2 pS27) was further confirmed by radioactive in vitro kinase assay by mixing GST‐AKT1, His‐Flag‐SOD2 WT, or phospho‐deficient His‐Flag‐SOD2 S27A, in which S27 was mutated to alanine (A), showing that AKT1 phosphorylated SOD2 WT while failed to phosphorylate SOD2 S27A (Figure 6C). However, compared to SOD2 WT, SOD2 S27A exhibited higher enzymatic activity in confined A549 cells (Figure 6D). These results suggest that AKT1 phosphorylates SOD2 S27 and thereby inhibits its enzymatic activity in confined cells.

To further confirm that SOD2 pS27 is regulated by IGFBP1 in confined cells, we generated a custom‐designed antibody specifically against SOD2 pS27 (anti‐SOD2 pS27). The specificity of the antibody was validated by immunoblotting analysis with this antibody of immunoprecipitated Flag‐SOD2 WT or S27A from HEK293T cells, showing that S27A mutation abrogated SOD2 pS27 (Figure S6F, Supporting Information). We then performed immunoblotting analysis of SOD2 pS27 in confined A549 cells with or without IGFBP1 depletion, showing that IGFBP1 depletion increased SOD2 pS27 in confined tumor cells (Figure 6E).

### 2.8 SOD2 S27 Phosphorylation Increases Mitochondrial ROS Levels and Apoptosis in Confined Cells and Impairs Tumor Metastasis

To investigate the biological role of SOD2 pS27, we first examined mitochondrial ROS levels in SOD2‐depleted A549 cells rescued with rSOD2 WT or phosphorylation‐mimetic rSOD2 S27D, in which S27 was mutated to aspartic acid (D), upon spatial constraints (Figure S6G, Supporting Information), which showed that the expression of rSOD2 S27D markedly increased mitochondrial ROS levels in confined cells compared to that of rSOD2 WT (Figure 6F). Moreover, tumor cells rescued with rSOD2 S27D exhibited much more apoptotic cells than the cells rescued with rSOD2 WT in confinement (Figure 6G). However, mitochondrial ROS levels and cell apoptosis were not influenced by rescued expression of rSOD2 S27D in the cells under no confinement (Figure S6H,I, Supporting Information). Additionally, bioluminescence imaging of the mice implanted with SOD2‐depleted A549 cells rescued with rSOD2 WT or S27D and H&E staining of dissected lung tissues from these mice showed that the expression of rSOD2 S27D greatly attenuated lung metastasis compared to that of rSOD2 WT (Figure 6H,I).

### 2.9 IGFPB1 Promotes the Survival of Confined Cells and Tumor Metastasis by Inhibiting SOD2 S27 Phosphorylation

**Figure 7:**
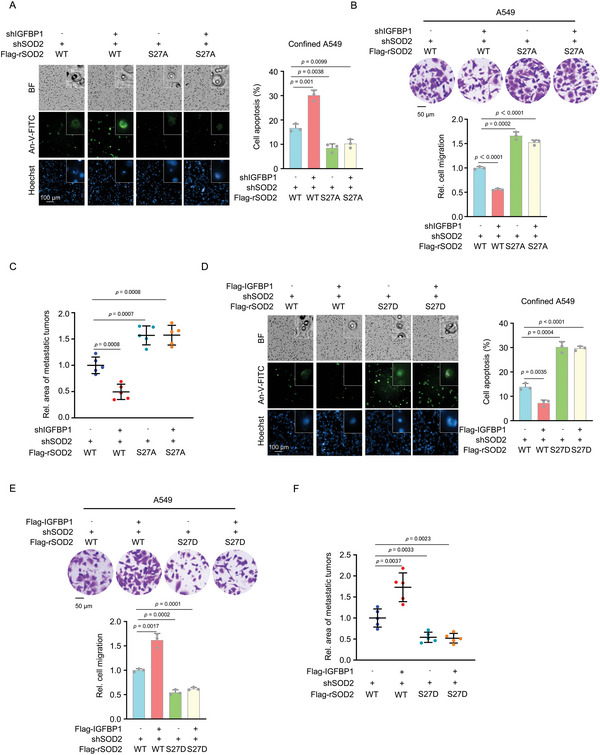
IGFPB1 promotes the survival of confined cells and tumor metastasis by inhibiting SOD2 S27 phosphorylation. A) IGFBP1 was depleted in SOD2‐depleted A549 cells rescued with rSOD2 WT or S27A. Transwell migration assays were performed. After 6 h of culture, the confined cells were stained with Annexin‐V‐FITC and photographed in situ. Representative images of the apoptotic confined cells were presented (left panel). The percentages of apoptosis of confined cells were shown (right panel). Data represent the mean ± S.D. of three independent experiments. B,C) IGFBP1 was depleted in SOD2‐depleted A549 cells rescued with rSOD2 WT or S27A. Transwell migration assays were performed (B). Representative images (top panel) and statistical analysis (bottom panel) of the migrated cells was shown. Data represent the mean ± S.D. of three independent experiments. These cells were implanted into randomized NOD/SCID mice by tail vein injection (five mice per group). After 30 days inoculation, the mice were euthanized. Tumor areas in H&E‐stained sections were calculated and normalized (C). Data represent the mean ± S.D. of five mice. D) IGFBP1 was overexpressed in SOD2‐depleted A549 cells rescued with rSOD2 WT or S27D. Transwell migration assays were performed. After 6 h of culture, the confined cells were stained with Annexin‐V‐FITC and photographed in situ. Representative images of the apoptotic confined cells were presented (left panel). The percentages of apoptosis of confined cells were shown (right panel). Data represent the mean ± S.D. of three independent experiments. E,F) IGFBP1 was overexpressed in SOD2‐depleted A549 cells rescued with rSOD2 WT or S27D. Transwell migration assays were performed (E). Representative images (top panel) and statistical analysis (bottom panel) of the migrated cells were shown. Data represent the mean ± S.D. of three independent experiments. These cells were injected into randomized NOD/SCID mice by tail vein injection (five mice per group). After 30 days inoculation, the mice were euthanized. Tumor areas in H&E‐stained sections were calculated and normalized (F). Data represent the mean ± S.D. of five mice.

To further confirm that IGFBP1 promotes tumor cell survival in confinement and subsequent metastasis by inhibiting SOD2 phosphorylation, we depleted IGFBP1 in SOD2‐depleted A549 cells rescued with rSOD2 WT or S27A (Figure S7A, Supporting Information) and found that IGFBP1 depletion dramatically increased the apoptosis and inhibited the migration of confined cells rescued with rSOD2 WT, while failed to do so in confined cells rescued with rSOD2 S27A (**Figure** **7**A,B). We also examined the apoptosis of these cells under no confinement and found no difference in cell apoptosis among these cells under no confinement (Figure S7B, Supporting Information). Moreover, we implanted these cells into randomized NOD/SCID mice by tail vein injection and found that IGFBP1 depletion dramatically inhibited the metastasis of tumor cells rescued with rSOD2 WT, while failed to do so in the cells rescued with rSOD2 S27A, as determined by H&E staining of dissected lung tissues (Figure 7C).

In addition, we overexpressed IGFBP1 in SOD2‐depleted A549 cells rescued with rSOD2 WT or S27D (Figure S7C, Supporting Information) and found that IGFBP1 overexpression dramatically inhibited the apoptosis and promoted the migration of confined cells rescued with rSOD2 WT, while failed to do so in confined cells rescued with rSOD2 S27D (Figure 7D,E). We also examined the apoptosis of these cells under no confinement and found no difference in cell apoptosis among these cells under no confinement (Figure S7D, Supporting Information). Moreover, we also implanted these cells into randomized NOD/SCID mice by tail vein injection and found that IGFBP1 overexpression dramatically promoted the metastasis of tumor cells rescued with rSOD2 WT, while failed to do so in the cells rescued with rSOD2 S27D (Figure 7F).

Collectively, these results indicate that IGFBP1 supports the survival of confined cells and enhances confined migration of tumor cells and therefore promotes tumor metastasis by inhibiting SOD2 S27 phosphorylation.

## 3 Discussion

**Figure 8:**
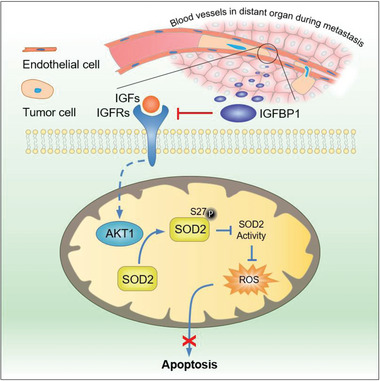
Schematic model for IGFBP1‐promoted tumor metastasis by sustaining cell survival during confined migration. IGFBP1 promotes tumor metastasis by sustaining cell survival during confined migration. Mechanistically, IGFBP1 enhances SOD2 activity by preventing AKT1‐mediated SOD2 S27 phosphorylation to scavenge accumulated mitochondrial ROS, thereby sustaining cell survival in confined space.

During metastatic cascade in vivo, cancer cells will navigate a complex spatially‐confined environment, including tracks along ECM fibers in the interstitial space, between muscle and nerve fibers, along or within blood vessels and in the vasculature of target organs. Cancer cells often switch their metabolism to anabolic pattern to support growth and proliferation, while during metastasis, which metabolic pathway is rewired and how rewired pathway promotes tumor metastasis remain largely unknown. In this study, we focused on metabolic regulation in tumor cells during confined migration and demonstrated that IGFBP1, a secreted protein, was upregulated in tumor cells during confined migration, which activated SOD2 to detoxify accumulated ROS in mitochondria by preventing AKT1‐dependent SOD2 phosphorylation, thereby sustaining the survival of confined cells and promoting tumor metastasis (**Figure** **8**). Our findings also underscore the therapeutic potential of neutralization of tumor‐secreted IGFBP1 for the patients with metastatic lung cancer as well as the diagnostic potential of IGFBP1 levels in blood.

IGFBPs are a family of serum proteins that bind to IGF‐I or II and regulate its turnover, transport, and tissue availability. IGFBP1 is only isoform with a tightly regulated expression and particularly important in regulating IGF‐I bioactivity, thereby modulating many of the physiological functions of IGFs, such as cell proliferation, survival, motility, and metabolism.^[^ ^23^ ^]^ In addition to its role in metabolic regulation, multiple studies indicate that IGFBP1 can inhibit tumor cell proliferation and motility in vitro in various human cancer cells and context.^[^ ^24^ ^]^ Meanwhile, there is also evidence that IGFBP1 can function as an oncogene, again in a context‐dependent manner, to promote tumor cell migration and drug resistance in vitro.^[^ ^25^ ^]^ However, the exact role of IGFBP1 in the locomotion through confining spaces, the important in vivo migration mode, and in tumor metastasis in vivo, and the underlying mechanism remain to be elucidated. In our study, we reveal the oncogenic role of IGFBP1 in lung cancer metastasis by supporting the survival of tumor cells during confined migration. Mechanically, IGFBP1 inhibits mitochondrial ROS accumulation induced by the confinement by activating SOD2, thereby supporting the survival of the confined cells. Importantly, the neutralization of secreted IGFBP1 inhibits the confined migration of tumor cells, while the treatment of recombinant IGFBP1 promotes the confined migration. In addition, immunohistological analysis of tumor tissues or ELISA analysis of blood samples from the patients with lung adenocarcinoma indicate that the levels of IGFBP1 positively correlate with the metastatic recurrence, suggesting the therapeutic and prognostic potential of IGFBP1 for metastatic lung cancer.

ROS plays important and complex roles in tumorigenesis and progression, from the regulation of signaling to the promotion of cell death, and therefore have both pro‐ and anti‐tumorigenic effects. To prevent excessive intracellular ROS, cancer cells respond to oxidative stress by inducing the transcription of antioxidant enzymes.^[^ ^26^ ^]^ Accumulating evidence show that a variety of physical force stimuli, such as cell attachment, cell stretching, shearing force and compression, will give rise to ROS,^[^ ^27^ ^]^ unevenly distributed in the cells,^[^ ^18^ ^]^ while how tumor cells cope with the accumulated ROS during confined migration remains to be elucidated. In present study, we demonstrate that IGFBP1 is upregulated in tumor cells during confined migration, which promotes ROS scavenging in mitochondria by activating SOD2, mitochondrial Mn‐SOD, thereby supporting the survival of tumor cells during confined migration. As mitochondria are the major place of ROS production, SOD2 is especially critical to cellular redox control.^[^ ^28^ ^]^

SOD2 has emerged as a key enzyme with a dual role in tumorigenic progression. SOD2 is traditionally regarded as a tumor suppressor in early studies. The reduction or loss of SOD2 expression has been shown to promote transformation and tumorigenesis by increasing ROS‐mediated DNA damage, as a consequence of the accumulation of superoxide anion free radical (O_2_•^−^) and oxidants generated from O_2_•^−^, such as ONOO^−^, H_2_O_2_, and HO• radical.^[^ ^29^ ^]^ In contrast to its suppressive role during tumor initiation, accumulating studies have shown that elevated SOD2 expression correlates with TMN stages and metastatic recurrence of various human cancers^[^ ^30^ ^]^ and is proven to be a necessary survival adaptation during metastasis, which enables cells to cope with increased cellular and extracellular redox stress.^[^ ^31^ ^]^ However, the regulatory mechanism underlying the activation of SOD2 during tumor metastasis in vivo, especially during confined migration, remains largely unknown. Here we demonstrate that SOD2 is phosphorylated by AKT1 at S27 and this phosphorylation inhibits the activity of SOD2. However, the upregulation of IGFBP1 in confined cells inhibits AKT1‐mediated S27 phosphorylation and therefore enhances the activity of SOD2. Although SOD2 has been shown to be phosphorylated at multiple residues, including S82 and S106,^[^ ^28^ ^]^ which residue is phosphorylated in cancer cells and what is the effect of the phosphorylation on SOD2 remain unknown. Our study identified a new phosphorylation of SOD2 at S27, which is removed in tumor cells during confined migration, thereby activating SOD2.

AKT kinase, the key component of the PI3K/AKT signaling pathway, regulates multiple cancer hallmarks, including the growth, proliferation, survival and migration of tumor cells. In mammals, the AKT kinase family includes three isoforms, AKT1, AKT2, and AKT3. Despite their high similarity, the distinct AKT isoforms exert non‐redundant, partly even opposing effects during cancer progression.^[^ ^32^ ^]^ Although numerous studies implicate a crucial role of PI3K‐AKT pathway in the regulation of cell motility, the role of AKT in cancer metastasis remains controversial. It has been shown that AKT1 activation promotes development of melanoma metastases.^[^ ^33^ ^]^ On the other hand, AKT1 has also been shown to inhibit cell migration and invasion by degrading the nuclear factor of activated T cells or abrogating *β*‐catenin signaling in human breast cancer cells^[^ ^34^ ^]^ or by reducing the phosphorylation of myristoylated alanine‐rich C‐kinase substrate and LAMC2 protein level in non‐small cell lung cancer cells.^[^ ^35^ ^]^ Additionally, activation of AKT1 can accelerate ErbB‐2‐mediated mammary tumorigenesis but suppresses tumor invasion.^[^ ^36^ ^]^ Similarly, in this study, we found that the activation of mitochondrial AKT1 inhibits tumor cell survival during confined migration, thereby impairing tumor metastasis. Mechanically, the activated AKT1 phosphorylates and inhibits SOD2 in mitochondria, thereby promoting mitochondrial ROS accumulation and subsequent cell death in confined tumor cells. AKT is well known to enhance malignancy and is recognized as a key target for antineoplastic therapies. However, our finding along with those of other researchers reveal that the inhibition of AKT1 also promotes tumor metastasis, which clearly justifies further investigations and calls for reevaluation of some AKT‐targeting therapeutic strategies currently under development.

## 4 Experimental Section

### Antibodies

Primary antibodies were used against: Flag (F1804, Sigma‐Aldrich); HA (3724, Cell Signaling Technology); IGFBP1 (ab181141, Abcam); phospho‐AKT T308 (13 038, Cell Signaling Technology); phospho‐AKT S473 (4060, Cell Signaling Technology); pan‐AKT (4685, Cell Signaling Technology); MEK1/2 (sc‐81504, Santa Cruz Biotechnology); phospho‐MEK1/2 (9154, Cell Signaling Technology); SOD2 (24127‐1‐AP, Proteintech); cytochrome c (4272, Cell Signaling Technology); COX IV (4850, Cell Signaling Technology); GST (SC‐138, Santa Cruz Biotechnology); tubulin (sc‐5267, Santa Cruz Biotechnology); *β*‐actin (3700, Cell Signaling Technology); phospho‐S6K T389 (9234, Cell Signaling Technology); phospho‐ERK1/2 (sc‐81492, Santa Cruz Biotechnology); and SOD2 pS27 (customized, Abclonal). The following secondary antibodies were used: goat‐anti‐mouse IgG second antibody (31160, Thermo); goat‐anti‐rabbit IgG second antibody (31210, Thermo); and goat anti‐rabbit Alexa Fluor Plus 647 (A32733, Thermo). The primary antibodies were used at a 1:1000 dilution for immunoblotting and a 1:200 dilution for IF and IHC. Secondary antibodies were used at 1:5000 dilution for immunoblotting and a 1:200 dilution for IF and IHC.

### Reagents

Flag peptide was obtained from Abclonal. Trizol was bought from Life Technologies. The Annexin V‐FITC/PI apoptosis detection kit was bought from BD Biosciences. The DeadEnd Fluorometric TUNEL System was bought from Promega. The SOD2 activity Assay Kit was bought from Beyotime Biotechnology. *N*‐acetyl‐l‐cysteine (A9165) was bought from Sigma‐Aldrich. PIEZO1 agonist (Yoda1), MEK1/2 inhibitor (PD0325901), and PI3K inhibitor (LY294002) were obtained from Selleckchem. EGTA Acetoxymethyl ester (HY‐D0973) was bought from MedChemExpress. D‐luciferin was bought from Xenogen. Ampicillin, kanamycin, puromycin, and hygromycin were bought from EMD Biosciences. In vitro DNA transfection reagent was purchased from Signagen Laboratories. [*γ*‐32P] ATP was bought from PerkinElmer. Recombinant full‐length human AKT1 was bought from SignalChem. Caspase‐Glo 3/7 Assay was bought from Promega.

### Cell Culture

All cell lines used were cultured at 37 °C, 5% CO_2_. A549 cells were maintained in RPMI‐1640 medium supplemented with 10% fetal bovine serum (FBS) and 1% penicillin–streptomycin. SK‐Hep1 and HEK293T cells were maintained in Dulbecco's modified Eagle's medium supplemented with 10% FBS and 1% penicillin–streptomycin. All cell lines were obtained from the type culture collection of the Chinese Academy of Sciences and validated by short tandem repeat (STR) testing.

### Animal Studies

6 weeks‐old female severe combined immune deficiency (SCID) mice were purchased from Lingchang Biotech (Shanghai, China). Littermates of the same sex were randomly assigned to experimental groups. All animal experiments were in accordance with ethical regulations and were approved by the institutional review board at the Institute of Biochemistry and Cell Biology.

### DNA Constructs and Mutagenesis

Human SOD2 was PCR‐amplified and cloned into pcDH/hygro (+)‐Flag, pColdI‐HIS vectors. Human IGFBP1, GPX4, TRXR2 were PCR‐amplified and cloned into pcDH/hygro (+)‐Flag. PCR‐amplified human AKT1 was cloned into pcDNA3.0/hygro (+)‐Flag. The mutations of SOD2 were made using the QuikChange II Site‐Directed Mutagenesis Kit. The pTRIPZ human IGFBP1 shRNA‐1 and shRNA‐2 were generated with 5′‐TCCATGTCACCAACATCAA‐3′ and 5′‐AGGAGACATCAGGAGAAGA‐3′ oligonucleotide. The pTRIPZ and pGIPZ human SOD2 shRNA‐1 was generated with 5′‐CAGCCTGCACTGAAGTTCA‐3′ oligonucleotide. The pLKO.1 human PIEZO1 shRNA‐1 was generated with 5′‐CTCACCAAGAAGTACAATCAT‐3′ oligonucleotide.

### Transwell Assays

Transwell assays (Corning BioCoat Control Insert‐No ECM, 8 µm pore size) were performed according to the manufacturer's instructions. Briefly, A549 (1 × 10^5^) or SK‐Hep1 (1 × 10^5^) cells were collected after trypsinization and added into Transwell chamber of 6.5 mm diameter with serum‐free medium. And medium with 10% FBS was used in the outside of the chamber. 12 h later, the cells were fixed by methanol and stained with 0.4% crystal violet. Non‐migrated cells on the upper side of the chamber were erased by a cotton swab. Migrated cells were photographed by inverted microscope and relative cell migration was calculated by the area of migrated cells and normalized to the control group. For each experiment, the area of migrated cells in more than three random fields (magnification, 200×) on the underside of the chambers was counted, and three independent experiments were analyzed.

### RNA‐Sequencing Samples Preparation

For RNA‐sequencing samples preparation, A549 (1 × 10^6^) cells were collected after trypsinization and added into Transwell chamber of 24 mm diameter (Corning BioCoat Control Insert‐No ECM, 8 µm pore size) with serum‐free medium. And medium with 20% FBS was used in the outside of the chamber. 18 h later, the membrane of chamber was immerged into trypsin and the cells adherent to the upper surface of the membrane and confined in the pore were collected separately. RNA of the collected cells was extracted with Trizol reagent (Life technologies).

### Wound Healing Assay

A549 cells (1 × 10^6^) were seeded in each well of a 6‐well culture plates. 12 h later, confluent monolayer of cells was scratched using a 200 µL pipette tip and photographs were taken for the first time 2 h after scratching when the cells on the board of the wound re‐adhered to the plates, this time point named as 0 h. Then the photographs of 24 h were taken. The relative migration of cells was calculated by measuring the width of the wounds and normalized to control groups.

### Quantitative Real‐Time PCR

Total RNA was extracted with Trizol reagent (Life Technologies). cDNA was prepared by HiScript II Q RT SuperMix for qPCR (+gDNA wiper) kit (Vazyme) and Quantitative Real‐Time PCR analysis was performed using ChamQ Universal SYBR qPCR Master Mix (Vazyme) under the following conditions: 5 min at 95 °C followed by 40 cycles at 95 °C for 30 s, 60 °C for 40 s, and 72°C for 1 min using a Roche LightCycler 96. The data of each experiment was normalized to the expression of control gene (*β*‐Actin) and represented in the manner of mean ± SD.

### Cell Proliferation Assay

A549 cells were seeded in 96‐well plates with 1000 cells per well and cultured with 200 µl complete culture medium containing 5% FBS. After 1, 3, and 5 days of culture, plates were collected and cells were fixed by trichloroacetic acid and stained by sulforhodamine B (SRB). OD560 values were determined by microplate reader. Relative cell proliferation was calculated by the OD560 values and normalized to day 1.

### Compression Experiments

Flexcell Compression system (Flexcell International Corporation) was utilized to apply the compressive load with a 0 or 5 kPa pressure, which were selected according to previous study,^[^ ^37^ ^]^ to A549 cells (1 × 10^6^) seeded on a glass slide covered with 1% agarose gel. After 12 h of culture, the IGFBP1 protein level was detected by immunoblotting analyses.

### H&E Staining

Lungs of the injected mice were removed and fixed with 4% PFA at 4 °C overnight. Then the lungs were embedded with paraffin. Each sample was entirely sectioned at an interval of 50 µm with a 5 µm thickness. For histologic observation, a standard H&E staining was performed.

### Immunofluorescence/TUNEL Staining

Lungs of the injected mice were removed and fixed with 4% PFA at 4°C overnight. Then frozen sections of the lungs were entirely sectioned at an interval of 50 µm with a 10 µm thickness. For histologic observation, a standard immunofluorescence staining was performed with anti‐CD31 antibody. The apoptotic cells in lungs were examined by TUNEL staining using the DeadEnd Fluorometric TUNEL System (Promega, G3250) following the manufacturer's protocol.

### Immunohistochemical Analysis

Paraffin‐embedded tissue sections of human lung cancer patients were stained with anti‐IGFBP1 antibody. the tissue sections were quantitatively scored according to the percentage of positive cells and staining intensity. The intensity of staining for a scale of 0–3 was rated: 0, negative; 1, weak; 2, moderate; and 3, strong. The following proportion scores were assigned: 0 if 0% of the tumor cells showed positive staining, 1 if 0–1% of cells were stained, 2 if 2–10% were stained, 3 if 11–30% were stained, 4 if 31–70% were stained, and 5 if 71–100% were stained. The proportion and intensity scores were then multiplied to obtain a total score. Scores were compared between the patients with metastatic recurrence and the patients without metastatic recurrence. The use of human lung tumor specimens and the database was approved by the Institutional Review Board at Fudan University Shanghai Cancer Center.

### ELISA Assay

ELISA studies were performed on plasma using Human IGFBP‐1 ELISA Kit (RayBio, ELH‐IGFBP1) and measured on microplate reader capable of measuring absorbance at 450 nm. The experimental procedure follows the manufacture's instruction.

### Purification of Recombinant Proteins

Flag‐tagged IGFBP1 wild‐type (WT) and SOD2 WT and S27A were cloned into pColdI‐HIS vector and expressed in bacteria and purified as described previously.^[^ ^38^ ^]^ Briefly, the vectors expressing Flag‐tagged IGFBP1 WT or SOD2 WT or SOD2 S27A were used to transform BL21/DE3 bacteria and proteins expressions were induced by adding 0.5 mm IPTG for 24 h at 16°C. BL21/DE3 cells were collected and sonicated in cold PBS containing proteasome inhibitors, followed by centrifugation at 14 000 rpm for 60 min (4 °C). The supernatants of the lysate were incubated with Ni‐NTA resin (Genescript) overnight at 4 °C on a rotator. Beads were washed three times before eluting with PBS (pH 7.4) containing 0.5 m imidazole and protein purities were verified by gel staining with Coomassie Blue.

### In Vitro Kinase Assay

The phosphorylation of SOD2 WT and S27A by AKT1 were demonstrated by in vitro kinase assay. In brief, bacterial‐purified SOD2 WT (2 µg) or S27A (2 µg) was incubated with AKT1 (0.5 µg, Signalchem) in kinase buffer (20 mm tris‐HCl, pH 7.2, 2.5 mm MnCl_2_, 2.5 mm MgCl_2_, 0.5 mm CaCl_2_, 1 mm DTT, 25 µm unlabeled, or 10 µCi *γ*‐^32^P labeled ATP) in a total volume of 50 µL at 37 °C for 30 min. Reactions were stopped by adding SDS‐PAGE loading buffer and the samples were subjected to SDS‐PAGE and detected by ^32^P autoradiography.

### SOD2 Enzyme Activity Assay

After 6 h of culture in Transwell (5 × 10^4^ cells), the cells were washed with PBS and treated with hypotonic buffer (10 mm tris‐HCl, 10 mm KCl, 1.5 mm MgCl_2_, pH 7.5) for 10 min and centrifuged for 10 min with 12000 × *g*. Then the collections were lysed by non‐contact sonication. After sonication, the lysis was centrifuged for 10 min with 12 000 × *g*, the supernatants were collected. SOD2 enzyme activity was determined using the corresponding detection kit (CuZn/Mn‐Superoxide Dismutase Assay Kit with WST‐8, Beyotime, S0103) according to the manufacture's instruction.

### Caspase‐3/7 Enzyme Activity Assay

After 6 h of culture in Transwell (5 × 10^4^ cells), the cells were washed with PBS and treated with hypotonic buffer (10 mm tris‐HCl, 10 mm KCl, 1.5 mm MgCl_2_, pH 7.5) for 10 min. Then the chambers were inserted into 1.5 mL Eppendorf tubes and centrifuged for 10 min with 12 000 × *g* and the supernatants were collected. For cells cultured in dishes, the cells were resuspended in hypotonic buffer, after trypsinizing and PBS washing, for 10 min and broken by grinding with a grinder (Bio Vision). Then the cell lysates were centrifuged for 10 min with 12 000 × *g* and the supernatants were collected. Finally, the activities of caspase‐3/7 in the collected supernatants were detected with the caspase‐3/7 activity detection kit of Promega following the manufacture's instruction.

### Tail Vein Injection

Cells were injected into tail vein of NOD/SCID mice at a dose of 2 × 10^6^ cells in 100 µL PBS per mouse. Right before tail vein injection, the mice were intraperitoneally injected with 40 mg kg^−1^ doxycycline. Then the doxycycline was injected every 3 days in the same way and with the same dose. Bioluminescence imaging was carried out (IVIS, Xenogen) at indicated time points. After the bioluminescence imaging, the mice were euthanized and dissected. The lungs were taken out for 4% PFA fixation and embedded for H&E or IF staining.

### Bioluminescence Imaging with IVIS

100 µL of 7.5 mg mL^−1^ d‐luciferin (Xenogen) was intraperitoneally injected into each mouse (five mice per group). Mice were anaesthetized with isoflurane inhalation. Bioluminescence imaging with a CCD camera (IVIS, Xenogen) was initiated 10 min after d‐luciferin injection with 5 min exposure time. Bioluminescence from the region of interest was defined manually. Background was defined using a region of interest from a mouse that was not given an intraperitoneal injection of d‐luciferin. All bioluminescent data were collected and analyzed using IVIS.

### Immunoprecipitation and Immunoblotting Analysis

Extraction of proteins with a modified buffer from cultured cells was followed by immunoprecipitation and immunoblotting with corresponding antibodies, as described previously.^[^ ^38^ ^]^

### In Situ Cell Apoptosis Detection

After 6 h of culture in Transwell (5 × 10^4^ cells), the cells were stained with Annexin‐V‐FITC kit (BD Biosciences) following the manufacturer's instruction. The pictures were taken by PerkinElmer Operetta high‐content imager and Harmony software. In situ cell apoptosis percentages were calculated as the number of Annexin‐V‐FITC positive confined cells divided by the number of confined cells.

### Intracellular ROS Detection

ROS was detected using RoGFP sensors, which were constructed as previously described.^[^ ^18^ ^]^ The sensors exhibited reciprocal GFP intensity changes in response to changes in redox conditions and were expressed in mitochondria matrix (Mito‐RoGFP) or cytosol (Cyto‐RoGFP) respectively. The ROS states of mitochondria and cytosol in target cells were detected by PerkinElmer Operetta high‐content imager and Harmony software to determine the changes.

### Mass Spectrometry Analysis

In vitro kinase assays of GST‐tagged AKT1 and Flag‐tagged SOD2 WT or S27A were done, unlabeled ATP was used in the reactions. Then the reactions were terminated by freezing and processed for mass spectrometry. In brief, proteins were precipitated with acetone and the protein pellets were dried by using a SpeedVac for 1–2 min. The pellets were subsequently dissolved in 8 m urea, 100 mm tris‐HCl, pH 8.5. 5 mm TCEP (Thermo Scientific), and 10 mm iodoacetamide (Sigma) and alkylation were added to the solution and incubated at room temperature for 30 min, respectively. The protein mixtures were diluted four times and digested overnight with Trypsin at 1:50 w/w (Promega). The digested peptide solutions were desalted using a MonoSpin C18 column (GL Science, Tokyo, Japan) and dried with a SpeedVac.

The peptide mixtures were analyzed by LC/tandem MS (MS/MS). Data‐dependent tandem mass spectrometry (MS/MS) analysis was performed with a Q Exactive Orbitrap mass spectrometer (Thermo Scientific, San Jose, CA). The acquired MS/MS data were analyzed against a Swiss‐Prot *Homo sapiens* database using PEAKS Studio 8.5 (Bioinformatics Solutions, Waterloo, Ontario, Canada). The database search parameters were set as the followings: MS and MS/MS tolerance of 20 ppm and 0.1 Da, respectively, FDR was set as 1% and protein identification threshold was set as (−10 logP) ≧ 20.

### Materials Availability

This study did not generate new reagents.

### Statistics and Reproducibility

Statistical analyses were performed using Microsoft excel and GraphPad Prism software (v.8.0) and quantitative data were presented as the mean ± SD. To represent the results as a heat map, the quantitated minimum and maximum values were shown with the lowest and highest color intensity, respectively, and the remaining values were ranked according to their relative values. Statistical significance of the correlation of IHC staining between proteins in human lung adenocarcinoma was determined using two‐tailed Student's *t*‐test. *p* < 0.05 was considered to be significant (*p < 0.05, **p < 0.01). ROC curve and area under the curve analyses were applied to detect the optimal cutoff point that yielded the highest total accuracy for discriminating metastasis and non‐metastasis.

## Conflict of Interest

The authors declare no conflict of interest.

## Author Contributions

G.C., Y.Q., and P.W. contributed equally to this work. This study was conceived by W.Y.; W.Y. and G.C. designed the study. G.C. and Y.Q. performed most of the experiments and also contributed to data analyses and figure editing. P.W., X.Q., and F.Y. provided experimental assistance and provided reagents and pathological assistance. H.G. assisted in reviewing and editing the manuscript. C.X. and Y.Z. contributed to the conception and experimental design during revision. W.Y. wrote the manuscript with comments from all authors.

## Supporting information

Supporting Information

## Data Availability

The data that support the findings of this study are available from the corresponding author upon reasonable request.
