# Supplementary material for: IGFBP1 Sustains Cell Survival during Spatially‐Confined Migration and Promotes Tumor Metastasis
Source: Adv Sci (Weinh). 2023 Jun 9;10(21):2206540. doi: 10.1002/advs.202206540 (PMC10375137; doi:10.1002/advs.202206540)
Supplement: Supplementary file 1 — Supporting Information [file ADVS-10-2206540-s001.pdf]

ADVANCED SCIENCE
Open Access

# Supporting Information

for *Adv. Sci.*, DOI 10.1002/advs.202206540

IGFBP1 Sustains Cell Survival during Spatially-Confined Migration and Promotes Tumor Metastasis

*Guoqing Cai, Yijun Qi, Ping Wei, Hong Gao, Chenqi Xu, Yun Zhao, Xiujuan Qu*, Feng Yao* and Weiwei Yang**

# Supporting Information

## IGFBP1 Sustains Cell Survival during Spatially-Confined Migration and Promotes Tumor Metastasis

Guoqing Cai, Yijun Qi, Ping Wei, Hong Gao, Chenqi Xu, Yun Zhao, Xiujuan Qu*, Feng Yao *, Weiwei Yang *

## Supplemental figure legends

## Figure S1.

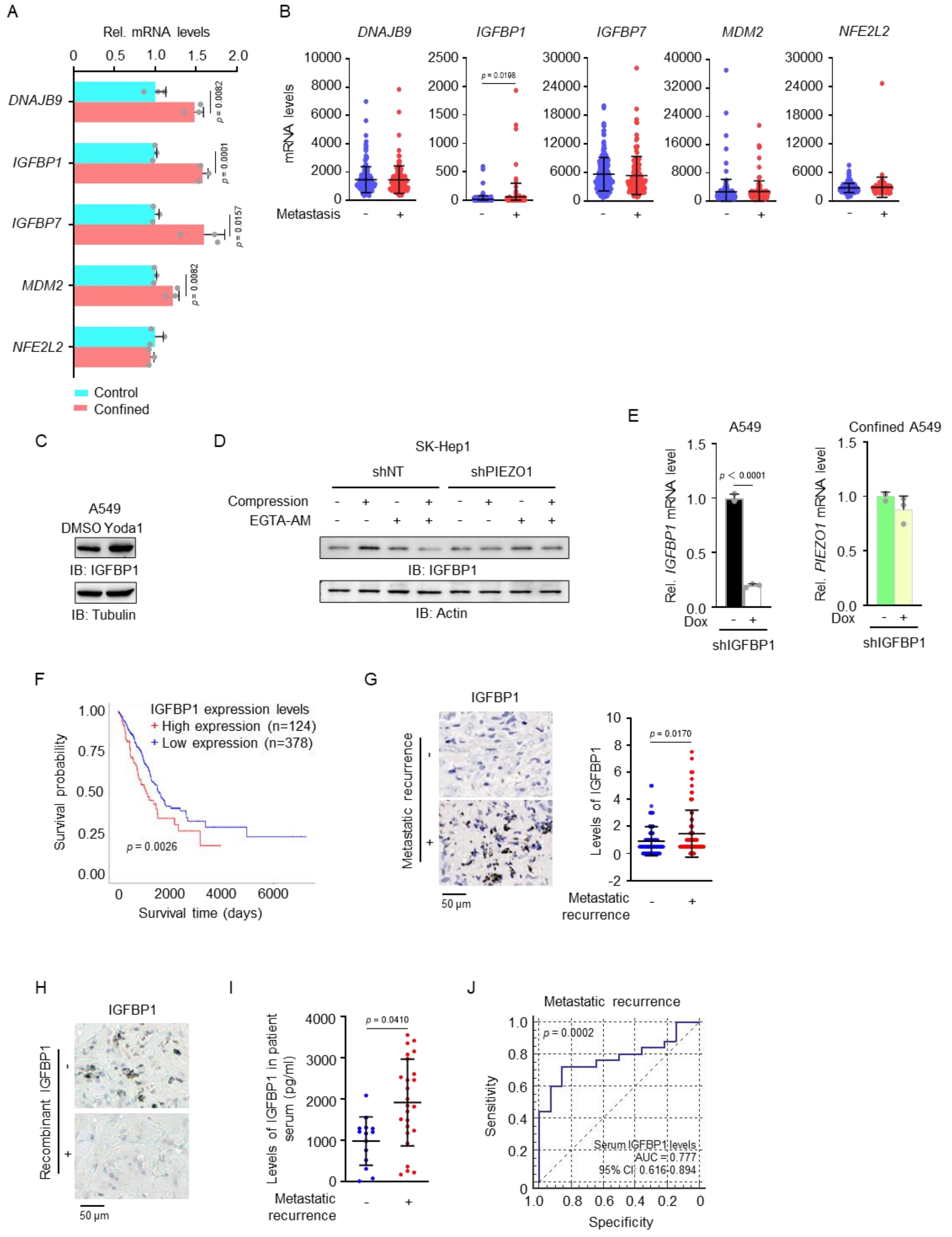

**Figure S1. IGFBP1 expression correlates with metastatic recurrence and prognosis of lung cancer patients, related to Figure 1**

(A) The mRNA levels of the top 5 overlapped genes in Fig. 1c, including *DNAJB9*, *IGFBP1*,

*IGFBP7*, *MDM2*, *NFE2L2*, were validated by quantitative PCR analysis.

(B) The mRNA levels of *DNAJB9*, *IGFBP1*, *IGFBP7*, *MDM2*, and *NEF2L2* were compared in patients with or without metastatic recurrence by using TCGA-LUAD dataset.

(C) IGFBP1 protein levels in A549 cells with or without Yoda1 treatment were detected by immunoblotting analyses.

(D) SK-Hep1 cells with or without PIEZO1 depletion were treated with or without EGTA-AM (30 μM) in the absence of presence of compression. IGFBP1 expression was examined by using immunoblotting analyses.

(E) The levels of *IGFBP1* and *PIEZO1* mRNA in confined A549 cells with or without IGFBP1 depletion were examined by quantitative PCR analysis.

(F) Survival durations of 502 patients with lung adenocarcinoma with low (n = 378, blue curve) or high (n = 124, red curve) expression of *IGFBP1* were compared (two-tailed log-rank test).

(G) Immunohistochemistry (IHC) staining of primary tumors from lung cancer patients with or without metastatic recurrence using anti-IGFBP1 antibody. Representative images are presented on the left panel. Staining scores of IGFBP1 were semiquantitatively analyzed and compared between the patients with (n=75) and without metastatic recurrence (n=90), statistical analysis is shown on the right panel.

(H) IHC analysis of IGFBP1 in tumor tissues from lung cancer patients with anti-IGFBP1 antibody were performed in the absence or presence of recombinant IGFBP1.

(I) The levels of IGFBP1 proteins in blood samples were compared between the patients with (n=25) and without metastatic recurrence (n=14) by using ELISA assay. Data represent the mean ± s.d. of the two groups.

(J) Receiver operating characteristic (ROC) analysis was performed with blood IGFBP1 levels in lung adenocarcinoma patients with or without metastatic recurrence. Area under the curve (AUC) and 95% confidence interval (CI) were presented.

**Figure S2.**

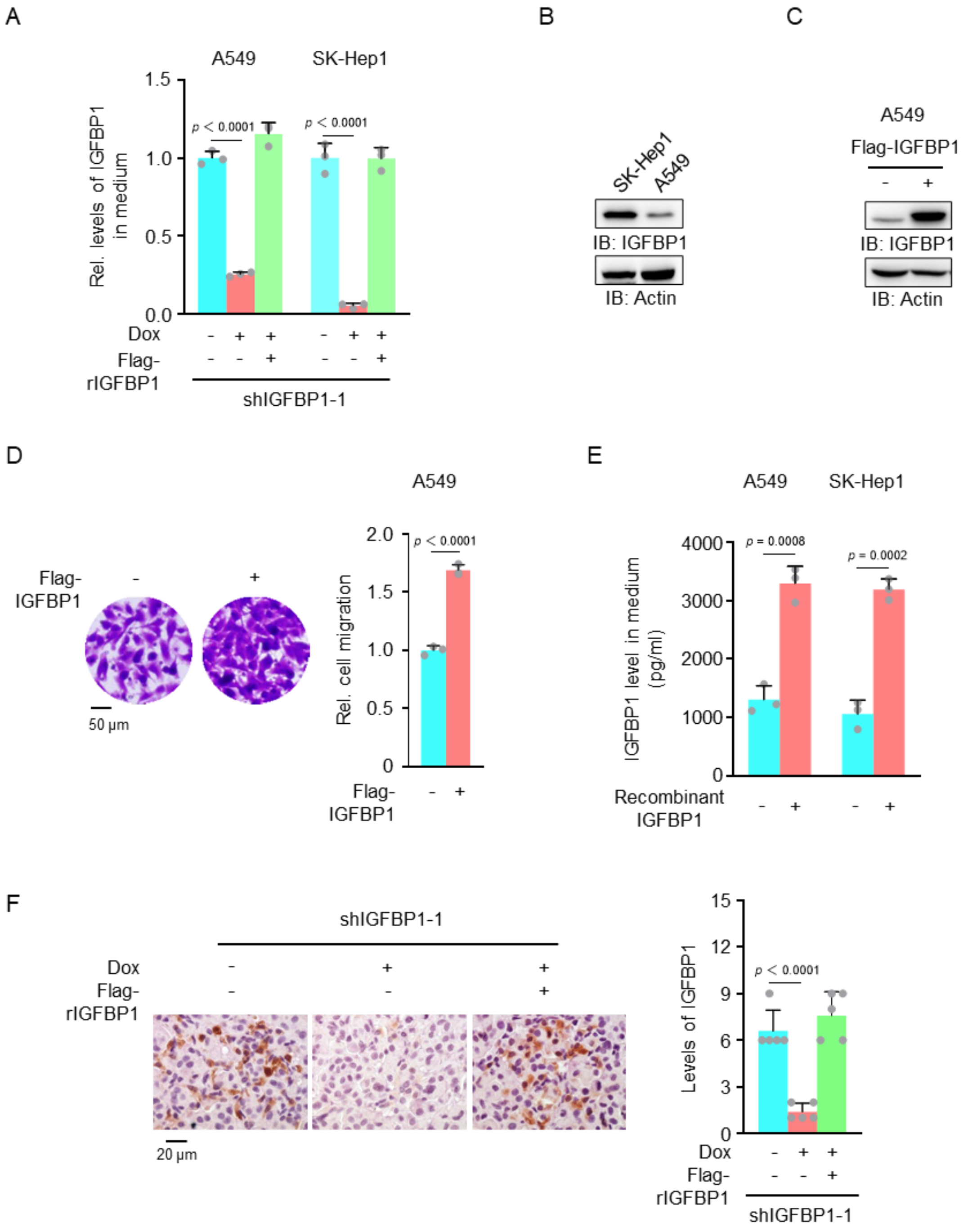


**Figure S2. The levels of IGFBP1 in medium and tissues, related to Figure 2**

(A) IGFBP1-depleted A549 and SK-Hep1 cells were rescued with or without Flag-rIGFBP1. Transwell migration assays were performed. Culture media of the cells were collected for the examination of secreted IGFBP1 by ELISA assay. Relative IGFBP1 levels of the medium were compared to those of A549 or SK-Hep1 cells without IGFBP1 depletion.

(B) IGFBP1 expression was examined in A549 and SK-Hep1 cells by using immunoblotting analyses.

(C) IGFBP1 expression was examined in A549 cells with or without IGFBP1 overexpression by using immunoblotting analyses.

(D) A549 cells were overexpressed with or without IGFBP1. Transwell migration assays were performed.

(E) A549 cells were cultured in Transwell chambers with 8 μm pore size and treated with or without 2500 pg/ml recombinant-IGFBP1. Total IGFBP1 levels of culture media were collected and detected by ELISA assay.

(F) Immunohistochemistry (IHC) staining of metastatic tumor in Figure 1H using anti-IGFBP1 antibody. Representative images are presented on the left panel. Staining scores of IGFBP1 were semiquantitatively analyzed and compared between the three groups. Representative images of IGFBP1 expression were shown on the left panel. Statistical analysis was shown on the right panel. Data represent the mean ± s.d. of five mice.

A, D, E, Data represent the mean ± s.d. of three independent experiments.

**Figure S3.**

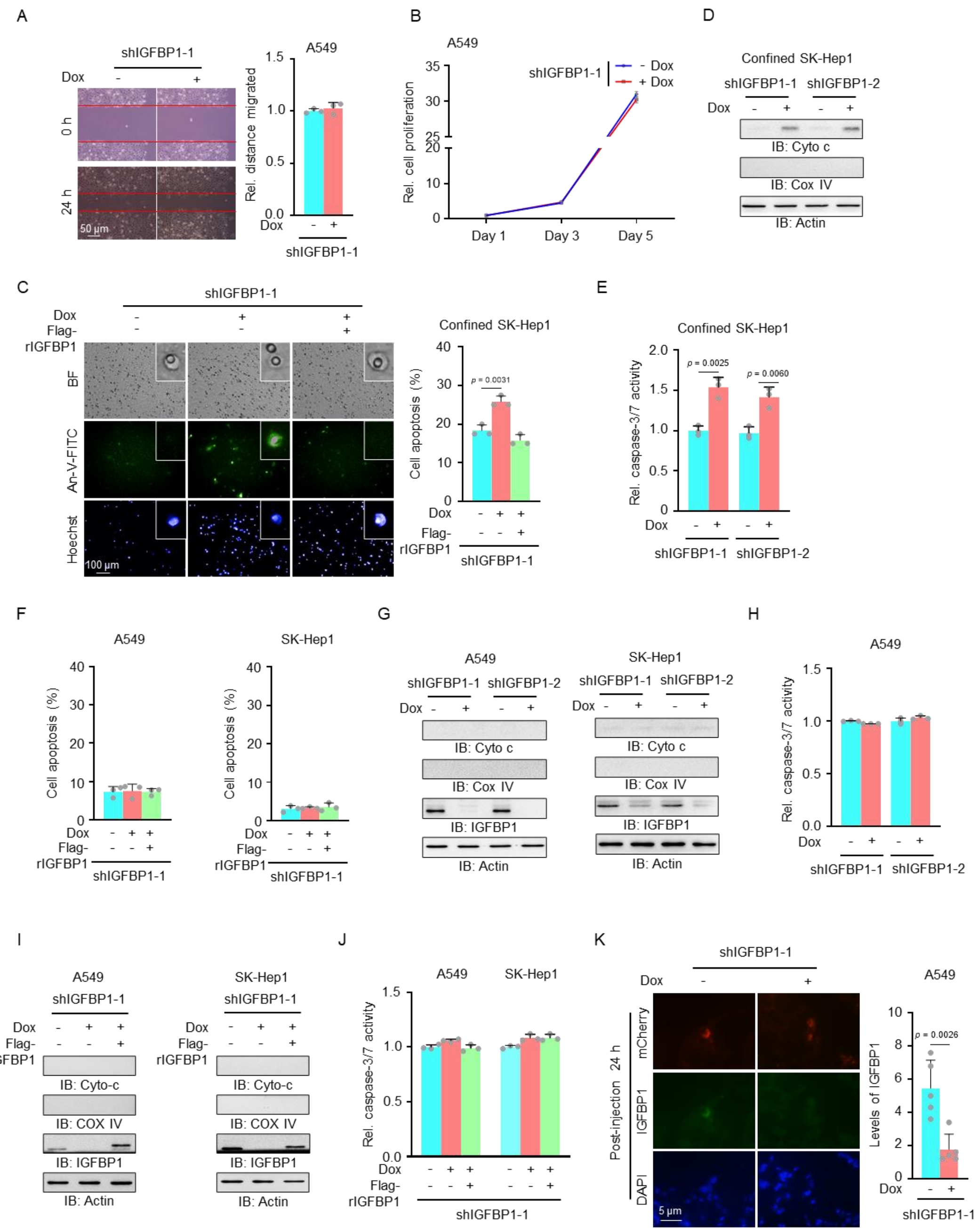


**Figure S3. IGFBP1 depletion did not influence cell migration, proliferation and apoptosis under normal condition, related to Figure 3**

(A) A549 cells with or without IGFBP1 depletion were seeded in 6-well plates. Wound healing assay was performed. Representative images of wound width changes were presented (left panel). Relative changes of wound width were normalized to those of A549 cells without

IGFBP1 depletion (right panel). Data represent the mean ± s.d. of three independen experiments.

(B) A549 cells with or without IGFBP1 depletion were seeded in 96-well plates. Cell proliferation assay was performed. Relative cell proliferation of A549 cells with or without IGFBP1 depletion was normalized to day 1 respectively. Data represent the mean ± s.d. of three independent experiments.

(C) IGFBP1-depleted SK-Hep1 cells were rescued with or without Flag-rIGFBP1. Transwell migration assays were performed. After 6 h of culture, the confined cells were stained with Annexin-V-FITC and photographed in situ. Representative images of the apoptotic confined cells were presented (left panel). The percentages of apoptosis of confined cells were shown (right panel). Data represent the mean ± s.d. of three independent experiments.

(D) Transwell migration assays of SK-Hep1 cells with or without IGFBP1 depletion were performed. After 6 h of culture, the confined cells were harvested and subjected to cell fractionation assay. The cytoplasmic fraction was used for immunoblotting analyses with indicated antibodies.

(E) Caspase-3/7 activities of confined SK-Hep1 cells were detected with the caspase-3/7 activity detection kit. Relative caspase-3/7 activities were compared to those of SK-Hep1 cells without IGFBP1 depletion. Data represent the mean ± s.d. of three independent experiments.

(F) IGFBP1-depleted A549 and SK-Hep1 cells were rescued with or without Flag-rIGFBP1. Cells were stained with Annexin-V-FITC and photographed in situ in 24-well plate. The percentages of confined cell apoptosis were shown. Data represent the mean ± s.d. of three independent experiments.

(G and H) A549 and SK-Hep1 cells with or without IGFBP1 depletion were harvested and subjected to cell fractionation assay. The cytoplasmic fraction was used for immunoblotting analyses with indicated antibodies (G). Caspase-3/7 activities in A549 cells were detected with the caspase-3/7 activity detection kit. Data represent the mean ± s.d. of three independent experiments (H).

(I and J) IGFBP1-depleted A549 and SK-Hep1 cells were rescued with or without Flag-rIGFBP1. These cells were subjected to cell fractionation assay. The cytoplasmic fraction was

used for immunoblotting analyses with indicated antibodies (I). Caspase-3/7 activities were examined in these cells. Relative caspase-3/7 activities were compared to those of A549 or SK-Hep1 cells without IGFBP1 depletion (J). Data represent the mean ± s.d. of three independent experiments.

(K) mCherry-expressing A549 cells with or without IGFBP1 depletion were injected into NOD/SCID mice via tail vein and lungs of the mice were dissected at 24 h after injection. IF staining with anti-IGFBP1 antibody of the lung sections were performed. Representative images of IGFBP1 expression were shown (left panel). Fluorescence intensity of IGFBP1 were calculated and shown (right panel). Data represent the mean ± s.d. of five mice.

**Figure S4.**

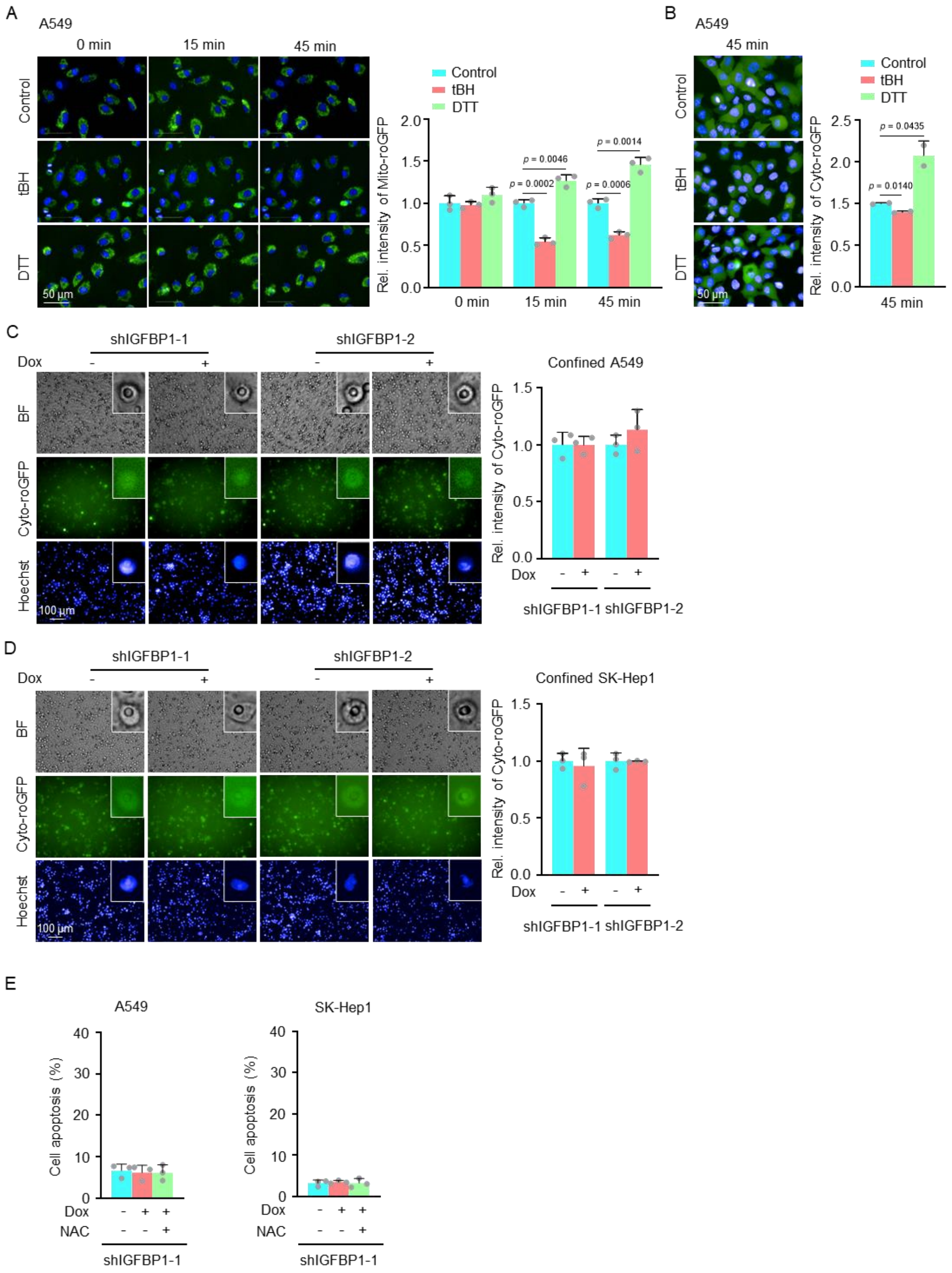


**Figure S4. IGFBP1 depletion did not influence the levels of cytosolic ROS in confined cells, related to Figure 4**

(A) Mito-roGFP-expressing A549 cells were seeded in 96-well plates and treated with or without 1 mM tBH or 1 mM DTT. The cells were photographed before treatment (0 min), 15

min and 45 min after treatment respectively. Representative images of Mito-roGFP were shown (left panel). Relative Mito-roGFP fluorescence intensities of each time point were normalized to those of the untreated Mito-roGFP-expressing A549 cells (right panel).

(B) Cyto-roGFP-expressing A549 cells were seeded in 96-well plates and treated with or without 1 mM tBH or 1 mM DTT. The cells were photographed 45 min after treatment and representative images of Cyto-roGFP were presented (left panel). Relative Cyto-roGFP fluorescence intensities were normalized to those of the untreated Cyto-roGFP-expressing A549 cells (right panel).

(C and D) Transwell migration assays of Cyto-roGFP-expressing A549 (C) or SK-Hep1 (D) cells with or without IGFBP1 depletion were performed. After 6 h of culture, the cells were photographed. Representative images of Cyto-roGFP in confined cells were presented on the left panel. Cyto-roGFP fluorescence intensities of confined cells were normalized to those of the Cyto-roGFP-expressing cells without IGFBP1 depletion and shown on the right panel.

(E) A549 and SK-Hep1 cells with or without IGFBP1 depletion were treated with or without NAC (5 mM). Cells were stained with Annexin-V-FITC and photographed in situ in 24-well plate. The percentages of confined cell apoptosis were shown.

A-E, Data represent the mean ± s.d. of three independent experiments.

**Figure S5.**

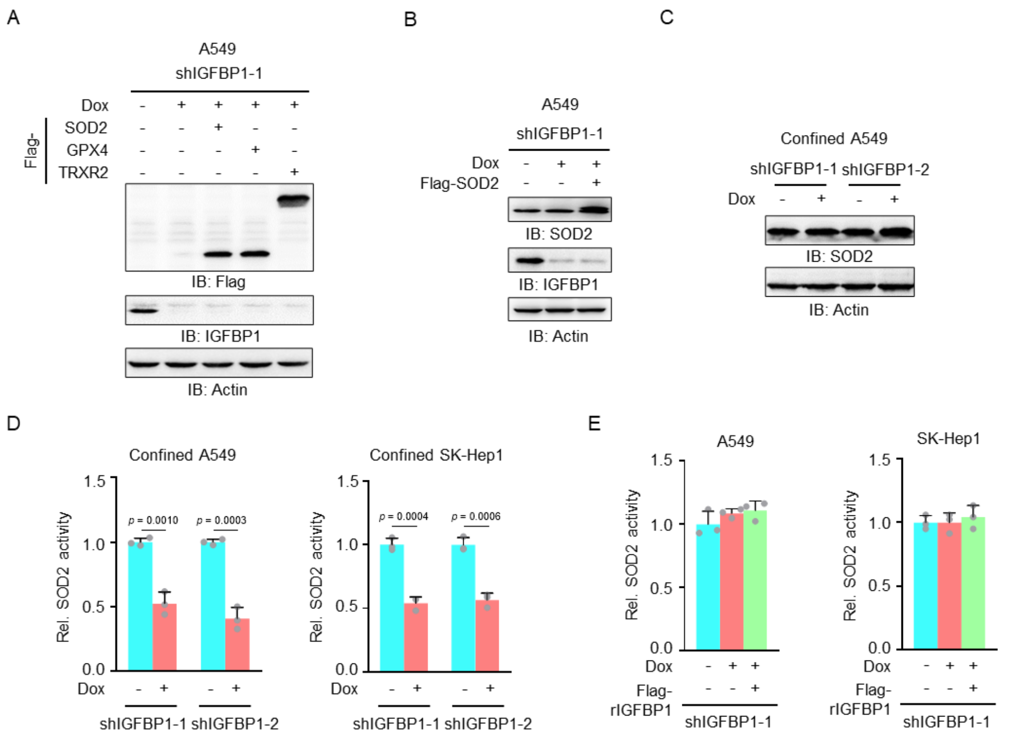


**Figure S5. IGFBP1 depletion inhibited SOD2 activities in confined cells, related to Figure 5**

(A) IGFBP-depleted A549 cells were overexpressed with empty vector (EV), Flag-SOD2, Flag-GPX4, or Flag-TRXR2. Immunoblotting analyses were performed.

(B) Flag-SOD2 was overexpressed in IGFBP1-depleted A549 cells. Control cells and IGFBP1-depleted cells with or without Flag-SOD2 overexpression were harvested for immunoblotting analysis with indicated antibodies.

(C) Transwell migration assays of A549 cells with or without IGFBP1 depletion were performed. After 6 h of culture, SOD2 protein levels in confined cells were detected by immunoblotting analyses with indicated antibodies.

(D) Transwell migration assays of A549 and SK-Hep1 cells with or without IGFBP1 depletion were performed. After 6 h of culture, SOD2 activities in confined cells were measured by WST-8 kit (Beyotime Biotechnology). Relative SOD2 activities were normalized to those of A549 or SK-Hep1 cells without IGFBP1 depletion. Data represent the mean ± s.d. of three independent experiments.

(E) IGFBP1-depleted A549 and SK-Hep1 cells were rescued with or without Flag-rIGFBP1. SOD2 activities of unconfined cells were measured by WST-8 kit (Beyotime Biotechnology). Relative SOD2 activities were normalized to those of A549 or SK-Hep1 cells without IGFBP1 depletion. Data represent the mean ± s.d. of three independent experiments.

**Figure S6.**

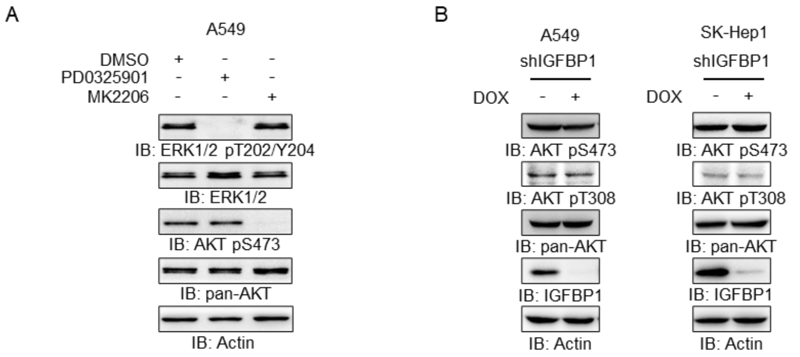


C

| Protein Accession | Peptide | PTM |
|---|---|---|
| P04179 | W.TNLS(+79.97)PNGGGEPKGELL.E | Phosphorylation**(S106)** |
| P04179 | L.EAIKRDFGS(+79.97)FDKFKEK.L | Phosphorylation**(S127)** |
| P04179 | L.EAIKRDFGS(+79.97)FDKF.K | Phosphorylation**(S127)** |
| P04179 | Q.KHS(+79.97)LPDLPY.D | Phosphorylation**(S27)** |
| P04179 | D.FGS(+79.97)FDKFKEKL.T | Phosphorylation**(S127)** |

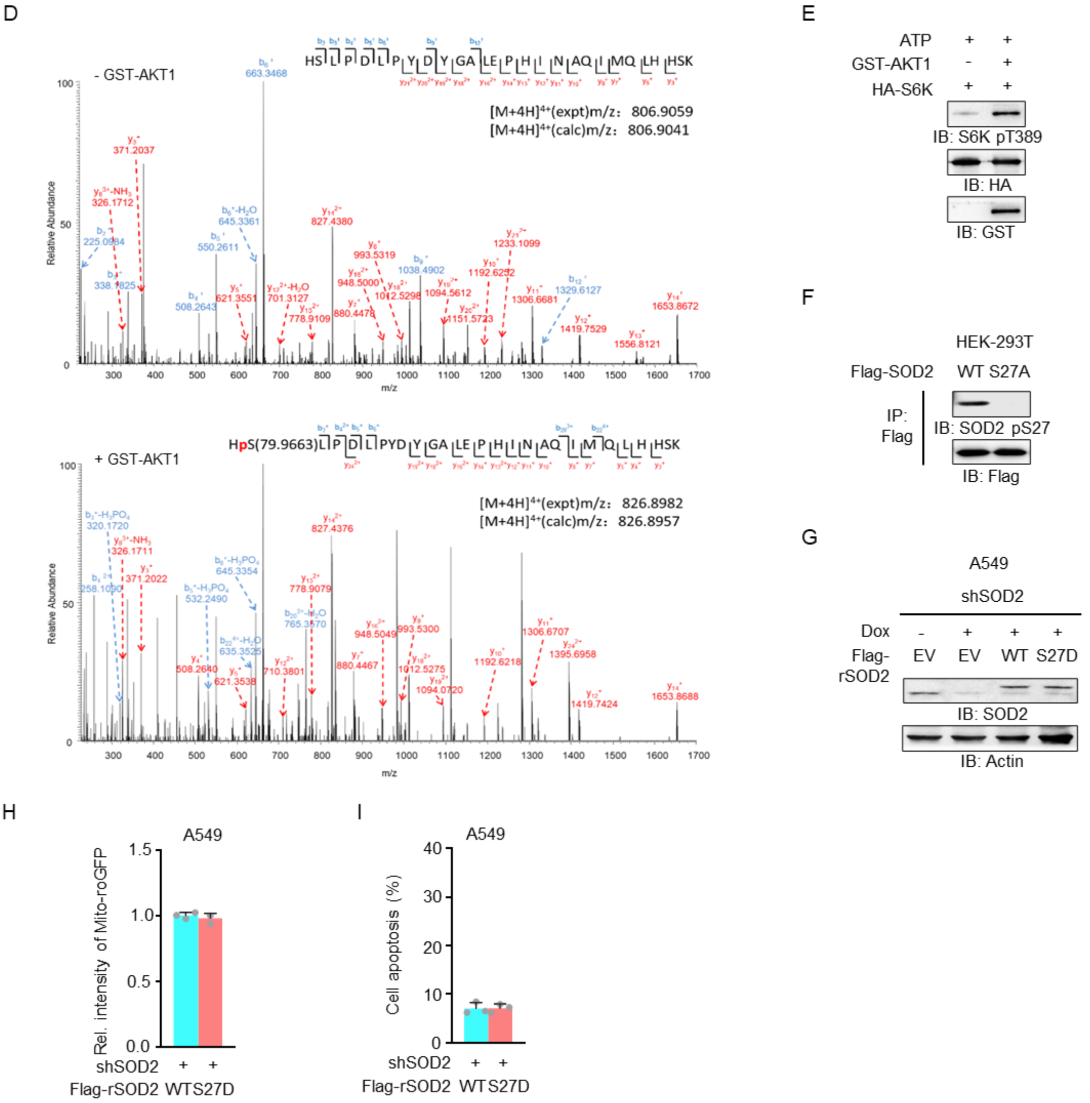

**Figure S6. AKT1 phosphorylates SOD2 at S27, related to Figure 6**

(A) A549 cells were treated with or without the inhibitor of MEK (PD0325901, 10 μM) or AKT1/2/3 (MK2206, 5 μM). Immunoblotting analyses were performed with indicated antibodies.

(B) A549 and SK-Hep1 cells with or without IGFBP1 depletion under no confinement were harvested for immunoblotting analyses with indicated antibodies.

(C) *In vitro* kinase assay was performed by mixing bacterial-purified recombinant GST-AKT1 and recombinant Flag-SOD2. Then the sample was subjected to mass spectrometry analyses. Mass spectrometry analysis indicated that 3 serine sites in SOD2, including S27, S106 and S127, could be phosphorylated.

(D) Mass spectrometry analysis of Flag-SOD2 in the *in vitro* kinase assay in (B) with (top panel) or without (bottom panel) incubation of recombinant GST-AKT1, and identified that a tryptic fragment at m/z 826.8982 (z=+4), matched to the charged peptide HsLPDLPYDYGALEPHINAQIMQLHHSK, indicating that SOD2 is phosphorylated at S27. The probability of S27 phosphorylation was 99.9%.

(E) In vitro kinase assay was performed by mixing recombinant GST-AKT1 and HA-S6K immunoprecipitated from HEK293T cells. Immunoblotting analyses were performed.

(F) WT and S27A mutant SOD2 were immunoprecipitated from HEK293T cells and were detected by immunoblotting analyses with custom-designed antibody specifically against SOD2 pS27.

(G) SOD2-depleted A549 cells were rescued with WT or S27D mutant SOD2. Immunoblotting analyses were performed.

(H) Mito-roGFP-expressing SOD2-depleted A549 cells were rescued with WT or S27D mutant SOD2. The Mito-roGFP intensities of unconfined cells were detected and normalized to those of the cells expressing rSOD2 WT. Data represent the mean ± s.d. of three independent experiments.

(I) SOD2-depleted A549 cells were rescued with WT or S27D mutant SOD2. The cells were stained with Annexin-V-FITC in situ. The percentages of apoptosis of unconfined cells were shown. Data represent the mean ± s.d. of three independent experiments.**Figure S7.**

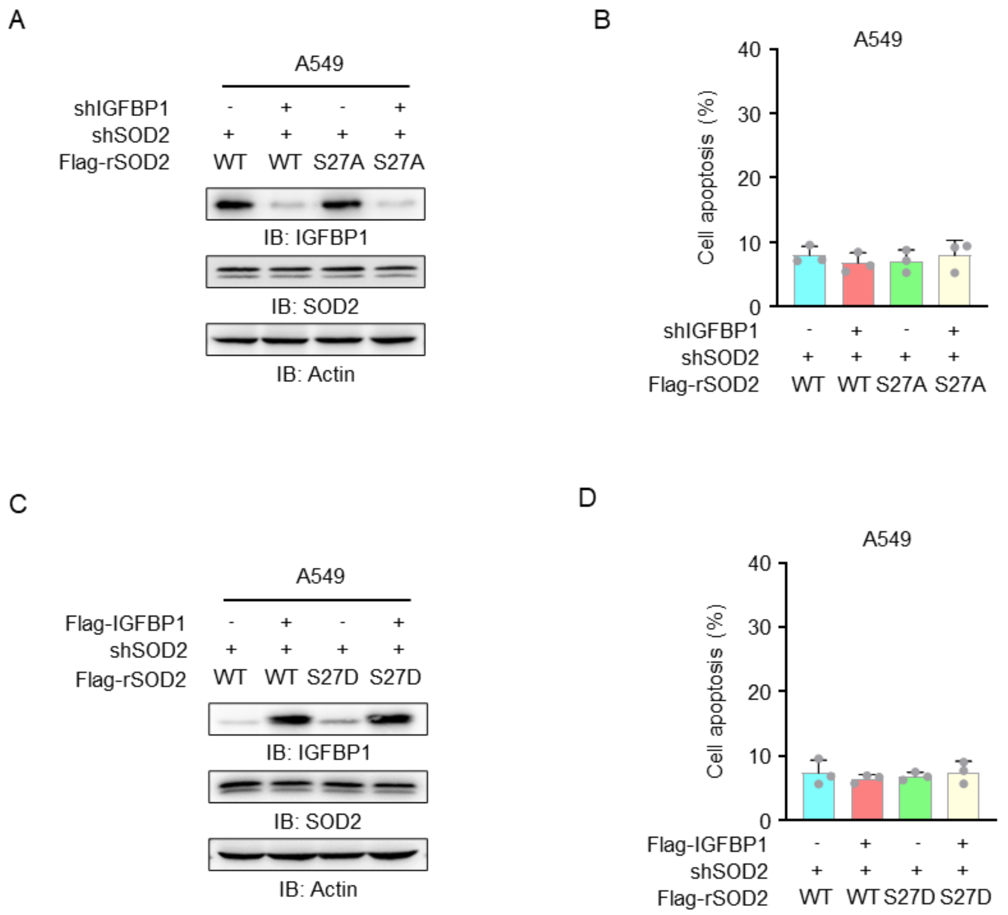


**Figure S7. IGFPB1-regulated SOD2 pS27 did not influence the survival of tumor cells under no confinement, related to Figure 7**

(A) IGFBP1 was depleted in SOD2-depleted A549 cells rescued with rSOD2 WT or S27A. Immunoblotting analyses were performed with indicated antibodies.

(B) IGFBP1 was depleted in SOD2-depleted A549 cells rescued with rSOD2 WT or S27A. The cells were stained with Annexin-V-FITC in situ. The percentages of apoptosis of unconfined cells were shown. Data represent the mean ± s.d. of three independent experiments.

(C) IGFBP1 was overexpressed in SOD2-depleted A549 cells rescued with rSOD2 WT or S27D. Immunoblotting analyses were performed with indicated antibodies.

(D) IGFBP1 was overexpressed in SOD2-depleted A549 cells rescued with rSOD2 WT or S27D. The cells were stained with Annexin-V-FITC in situ. The percentages of apoptosis of unconfined cells were shown. Data represent the mean ± s.d. of three independent experiments.
